# MiR396‐*GRF* module associates with switchgrass biomass yield and feedstock quality

**DOI:** 10.1111/pbi.13567

**Published:** 2021-02-24

**Authors:** Yanrong Liu, Jianping Yan, Kexin Wang, Dayong Li, Rui Yang, Hong Luo, Wanjun Zhang

**Affiliations:** ^1^ College of Grassland Science and technology China Agricultural University Beijing China; ^2^ College of Life Sciences Shandong Normal University Jinan Shandong China; ^3^ Beijing Key Laboratory of New Technology in Agricultural Application College of Plant Science and Technology Beijing University of Agriculture Beijing China; ^4^ Department of Genetics and Biochemistry Clemson University Clemson SC USA; ^5^ Key Lab of Grassland Science in Beijing China Agricultural University Beijing China

**Keywords:** biomass yield, feedstock quality, GRF, miR396, switchgrass

## Abstract

Improving plant biomass yield and/or feedstock quality for highly efficient lignocellulose conversion has been the main research focus in genetic modification of switchgrass (*Panicum virgatum* L.), a dedicated model plant for biofuel production. Here, we proved that overexpression of miR396 (OE‐miR396) leads to reduced plant height and lignin content mainly by reducing G‐lignin monomer content. We identified nineteen *PvGRFs* in switchgrass and proved thirteen of them were cleaved by miR396. MiR396‐targeted *PvGRF1*, *PvGRF9* and *PvGRF3* showed significantly higher expression in stem. By separately overexpressing *rPvGRF1*, *3* and *9*, in which synonymous mutations abolished the miR396 target sites, and suppression of PvGRF1/3/9 activity *via PvGRF1*/*3*/*9‐SRDX* overexpression in switchgrass, we confirmed *PvGRF1* and *PvGRF9* played positive roles in improving plant height and G‐lignin content. Overexpression of *PvGRF9* was sufficient to complement the defective phenotype of OE‐miR396 plants. MiR396‐*PvGRF9* modulates these traits partly by interfering GA and auxin biosynthesis and signalling transduction and cell wall lignin, glucose and xylan biosynthesis pathways. Moreover, by enzymatic hydrolysis analyses, we found that overexpression of *rPvGRF9* significantly enhanced per plant sugar yield. Our results suggest that *PvGRF9* can be utilized as a candidate molecular tool in modifying plant biomass yield and feedstock quality.

## Introduction

Switchgrass (*Panicum virgatum* L.), a warm season perennial species, has been recognized as a model plant of lignocellulosic material supplier for biofuel production (Peixoto and Sage, 2016; Sage et al., 2015). Improvement of biomass yield (Do et al., 2016; Fu et al., 2012) and/or lignocellulose conversion efficiency (Fu et al., 2011; Park et al., 2017; Srivastava et al., 2015; Xu et al., 2011) have been the main research focuses of switchgrass genetic improvement. However, a tricky trade‐off has always been noticed between them (Do et al., 2016; Fu et al., 2012; Sanford et al., 2017). Focusing on improving one of the traits also takes the risk of emerging undesirable effects on other traits, which may result in no net benefit in biofuel production (Li et al., 2010; Park et al., 2017; Ponniah et al., 2017; Shadle et al., 2007; Zhao and Dixon, 2014). Therefore, it is still necessary to explore the key regulatory pathways to identify potential targets for modification, achieving significantly high impact beneficial to overall plant growth without strong or obvious deleterious effect.

MicroRNA (miRNA), as one kind of the plant small non‐coding RNA, serves a powerful biological function in plant growth and development through modulating the mRNA abundance of its target genes and indirectly interfering with several plant hormonal signalling transduction pathways (Basso et al., 2019; Singh et al., 2018). The paradigmatic model of the miR396‐*GROWTH‐REGULATING FACTOR* (*GRF*) genes has been investigated and demonstrated to play vital roles in the control of the size of multiple plant tissues or organs, such as leaf (Liu et al., 2009; Mecchia et al., 2013), flower (Liu et al., 2014; Tang et al., 2018; Yuan et al., 2020) and root (Ercoli et al., 2016; Rodriguez et al., 2015). Modulating the expression level of miR396 or its targeted *GRF* genes has been proven the efficient strategies in improving plant agronomic traits. For example, overexpression of *OsGRF4* increased grain size (Li et al., 2018) and reduced leaf angle in rice (Tang et al., 2018). Repression of miR396 increased fruit size in tomato (Cao et al., 2016) while enhanced pathogen resistance in rice (Chandran et al., 2019). Besides, miR396‐*GRF* module negatively regulated the nematode infection and plant development in soybean (Noon et al., 2019). However, few reports focused on the biological function of miR396‐*GRF* regulatory pathway on plant height, biomass yield and feedstock quality of biofuel species.

Plant height and cell wall lignin content are the main factors affecting biomass yield and quality, respectively (Li et al., 2014). MiR396 has previously been implicated in negatively regulating stem elongation (Franco‐Zorrilla et al., 2007; Gao et al., 2015), and *GRF3/10* in rice played a positive role in regulating plant height (Kuijt et al., 2014; Tang et al., 2018). Further studies showed that overexpression of miR396d attenuates gibberellin (GA) biosynthesis and signalling transduction (Tang et al., 2018; Tong et al., 2014; Voorend et al., 2016; Wuddineh et al., 2015). Increasing GA level in switchgrass significantly improved plant height, but cell wall lignin content was also increased (Do et al., 2016; Wuddineh et al., 2015). These results imply that miR396‐*GRF* module may play opposite roles in regulating plant height, biomass yield and feedstock quality, and miR396 and *GRFs* seem to function synergistically to regulate plant growth and development (Ercoli et al., 2016; Rodriguez et al., 2010).

GRF family is one of the conserved plant‐specific transcription factor (TF) families and has been investigated in many plant species including *Arabidopsis* (Choi et al., 2004), rice (Choi et al., 2004) and maize (Zhang et al., 2008) characterized with two conserved domains: the QLQ domain and the WRC domain. GRF family in different species is generally composed of 8–20 members, most of which contain miR396 target sites (Omidbakhshfard et al., 2015). Different members of GRF family function redundantly and/or independently in regulating tissues development (Omidbakhshfard et al., 2015). For example, in *Arabidopsis*, *AtGRF1* to *AtGRF5* function redundantly in improving leaf size (Debernardi et al., 2014; Vercruysse et al., 2020), while *AtGRF9* plays a negative role in regulating leaf size (Omidbakhshfard et al., 2018). And the function of *AtGRF5* cannot be replaced by other family members (Horiguchi et al., 2005). Similarly, rice *OsGRF3* and *OsGRF10* genes have been reported to play a positive role (Kuijt et al., 2014; Tang et al., 2018), while *OsGRF1* and *OsGRF7* play a negative role in regulating plant height (Chandran et al., 2019; Van Der Knaap et al., 2000). These results suggest different *GRF* genes may perform opposite functions in regulating plant development. Therefore, instead of directly modulating the expression of miR396, modifying the expression of a functional specific *GRF* gene might be a more promising strategy for desirable traits (Li et al., 2018).

In switchgrass, the presence of miR396‐*GRF* regulatory pathway has been reported based on RNA sequencing data (Matts et al., 2017; Xie et al., 2014). We speculated that the miR396‐*GRF* module could play important roles in switchgrass biomass yield and feedstock quality. To this end, we improved expression of miR396 by introducing a rice *Osa‐MIR396a* gene in switchgrass and investigated the biological function of miR396 in modifying plant morphology and lignification. We proved that miR396 targets, *PvGRF1* and *PvGRF9*, but not *PvGRF3*, play important positive roles in promoting plant height and lignification. Overexpression of *PvGRF9* in OE‐miR396 plants was sufficient to restore the phenotype altered by miR396 overexpression. By analysing feedstock quality of wild type (WT) and different transgenic switchgrass plants, we found that overexpression of *rPvGRF9* leads to the highest sugar yield production. Our findings confirm that miR396‐*GRF* associates with switchgrass height and lignification, and *PvGRF9* plays dominant roles. This study suggests a promising avenue for improvement of switchgrass biofuel characteristics and genetic modification of other forage crops.

## Results

### Overexpression of miR396 leads to reduced plant height and biomass yield in switchgrass

**Figure 1:**
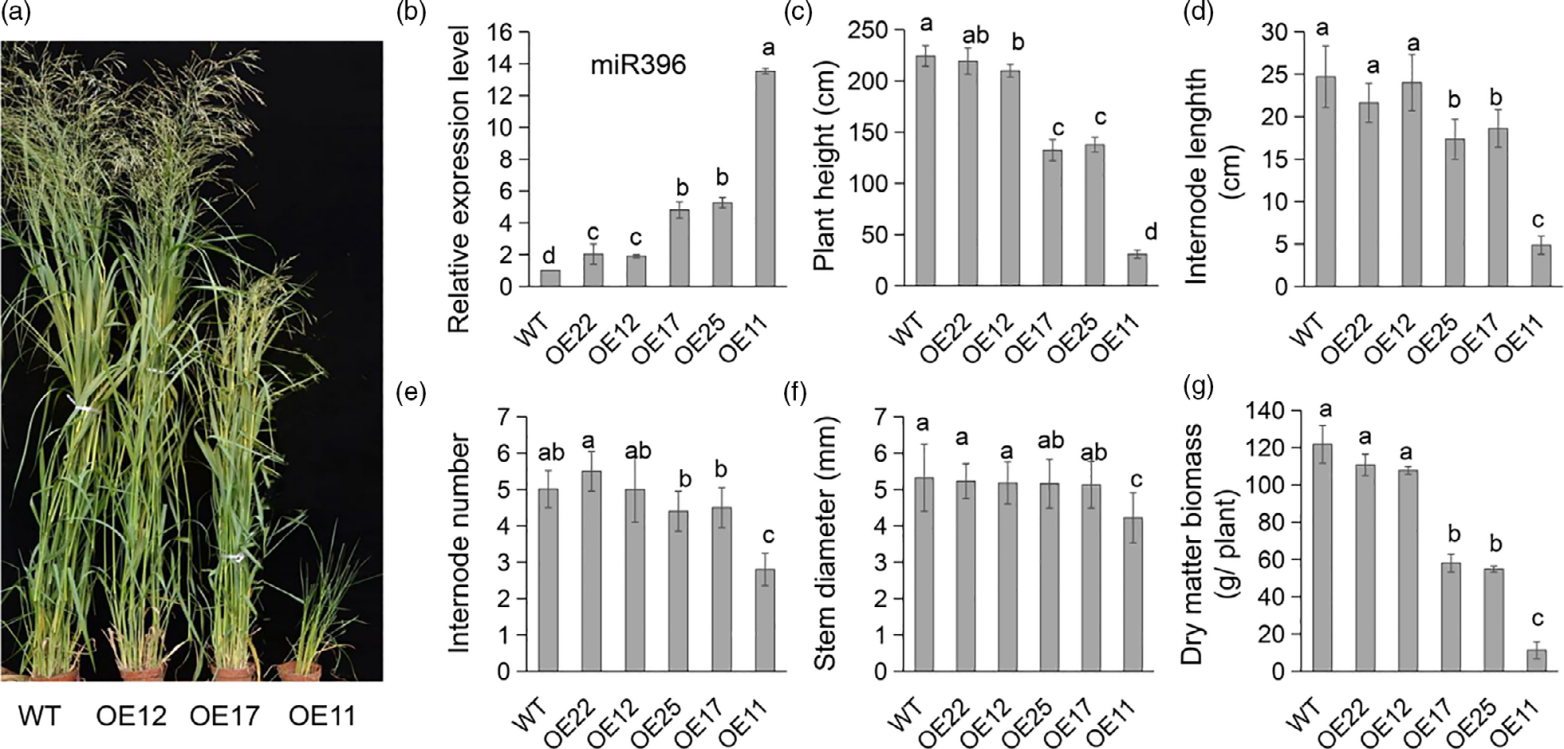
Phenotype comparison of the R3 stage switchgrass WT and OE‐miR396 plants. (a) Photograph of typical WT and OE‐miR396 plants. (b) Relative expression of miR396 revealed by quantitative RT‐PCR (*n* = 3). A nuclear switchgrass small RNA gene, *U6* was used as an internal control. (c) Comparison of plant height (*n* = 4). (d, e) The average internode length (d) and number (e) (*n* = 4). (f) The bottom internode diameter of stem (*n* = 4). The data shown in (c), (d), (e) and (f) are the means of four biological replicates (with twenty technical repeats each) ± SD. (g) Comparison of dry biomass yield of WT and OE‐miR396 plants (*n* = 4). The error bar indicates standard deviation. The different letters indicate statistically significant differences determined by Duncan’s multiple range test (*P* < 0.05).

Switchgrass miR396 (Pv‐miR396) has previously been reported as a result of miRNA sequencing (Matts et al., 2017; Xie et al., 2014) whose sequence is the same as that in *Arabidopsis*, At‐miR396a, and rice, Osa‐miR396a/b (Figure S1a). Pv‐miR396 was ubiquitously expressed in flower, stem and leaf tissues, and its expression level was significantly higher in mature florets than in stems and leaves (Figure S1b, c). To investigate the biological function of miR396 in switchgrass, we up‐regulated the expression level of miR396 by overexpression of *Osa‐MIR396a* (*GQ419538*) gene (Figure S2a). Twenty‐three independent transgenic lines were generated and confirmed by amplification of the *Osa‐MIR396a* gene with PCR and RT‐PCR (Figure S2b, c). Relative expression level of miR396 in WT and different *Osa‐MIR396a* transgenic lines was further tested by qRT‐PCR (Figure 1b). We classified the OE‐miR396 plants into three groups based on the expression level of miR396 and plant height (Figure 1a, b, Figure S2d). Five representative OE‐miR396 lines, OE12 and OE22 (normal height, 4/23), OE17, and OE25 (semi‐dwarf, 13/23), and OE11 (extremely dwarf, 6/23) were selected for further studies. The transgenic plants were grown in a glasshouse for six months to R3 stage (Hardin et al., 2013). The morphology of OE12 and OE22 was similar to that of WT. OE17 and OE25 was about half as tall as WT, and OE11 had the highest expression of miR396 with the shortest plant height (about 20% of WT) (Figure 1c). Detailed phenotypic analyses indicated that the reduction in plant height of the OE‐miR396 plants was due to the length reduction of internode (Figure 1d) and inflorescence compared to WT control plants (Table S1). Among them OE11 had a smallest internode number and stem diameter (Figure 1e, f). Under a scanning electron microscopy, we observed the stem cell size and length of OE17 were significantly smaller and shorter than that of WT controls (Figure S3a–d), while the long cell number of the first internode (top) of the E3 stage tiller (1NE3) had no significant difference between WT and OE17 (Figure S3e). Moreover, the leaf blade width and length of the transgenic plants also showed a significant reduction positively correlated to miR396 expression level, whereas no significant difference in tiller number between WT and OE‐miR396 plants was observed (Table S1). Taken together, miR396 overexpression alters plant development in OE17, OE25 and OE11 resulting in a significant reduction in plant biomass yield (Figure 1g).

### MiR396 negatively affected stem lignin and glucose content in switchgrass

**Figure 2:**
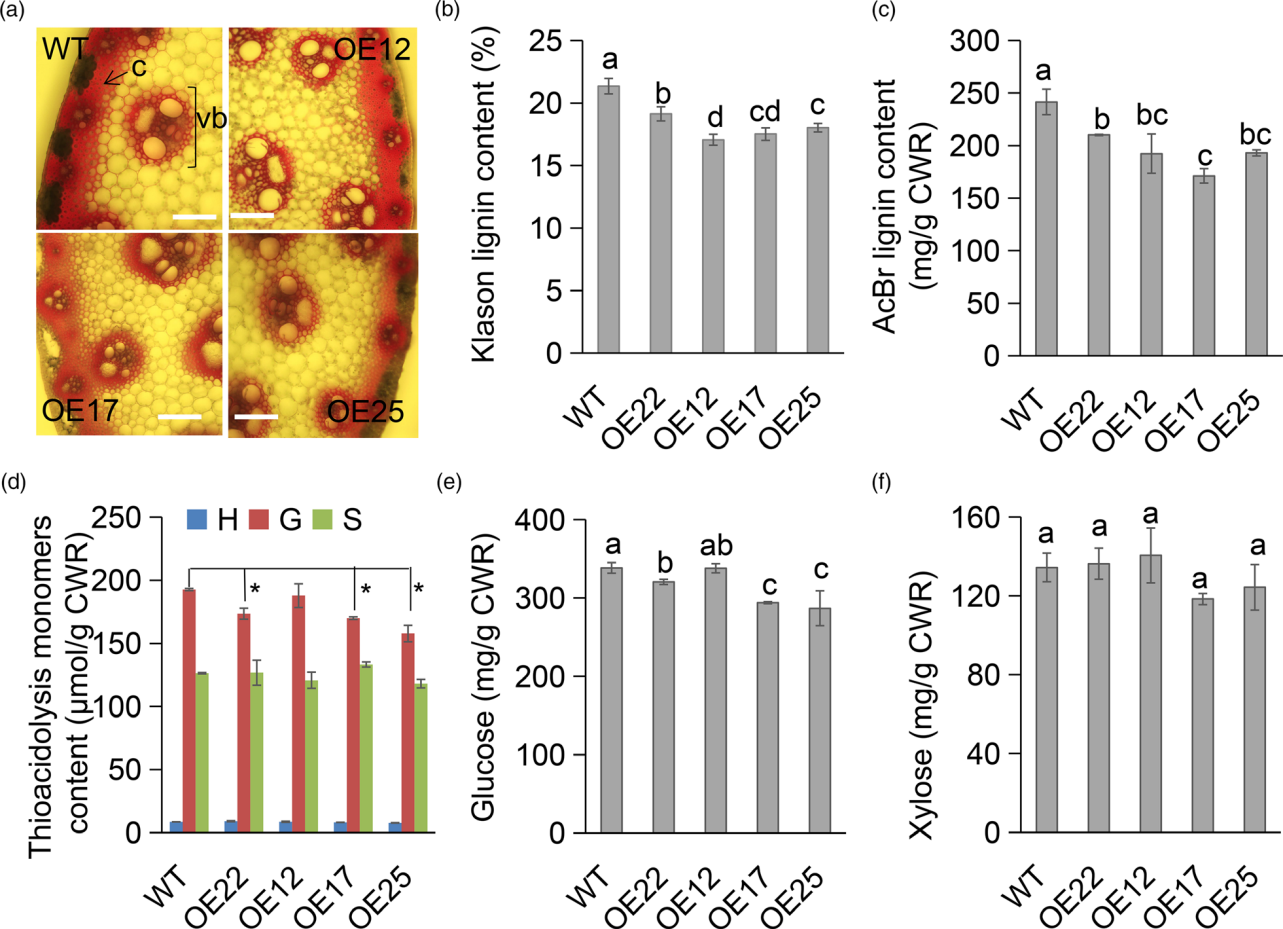
Stem cell wall components analysis of WT and OE‐miR396 lines. (a) Phloroglucinol‐HCl staining assay of lignin in 1NE3 cross sections of WT and OEs. The red coloration indicates the presence of lignin. c, collenchyma; vb, vascular bundle cells. Scale bar = 50 μm. (b) The Klason lignin of CWR of WT and OEs (*n* = 3) with five technical repeats each. (c, d) AcBr lignin content (c) and the lignin unit content (d) of CWR of WT and OEs (*n* = 2). G, guaiacyl; S, syringyl; H, *p*‐hydroxyphenyl. (e, f) The glucose (e) and xylose (f) content of CWR of WT and OEs (*n* = 3) with five technical repeats each. The error bar indicates standard deviation. Different letters and asterisks represent significant differences (*P* < 0.05).

To investigate the effects of miR396 on switchgrass stem cell wall composition, we first examined the lignin content of OE‐miR396 plants by histochemical staining. As shown in Figure 2a, the red coloration of the vascular bundle (vb) cells and collenchyma (c) cells of the OE‐miR396 plants was weaker than that of WT plants. Considering the uncertain reliability of different lignin assay methods, especially when there exist drastically different morphological characters among plant samples, we used two methods, Klason and acetyl bromide (AcBr) assays to measure total lignin content of the WT and transgenic lines. The results showed that the OE‐miR396 lines had significantly lower (approximately 15%) lignin content than WT controls (Figure 2b, c). Analysis of lignin monomers indicated that OE‐miR396 lines (except OE12) had significantly reduced lignin guaiacyl (G) unit content (Figure 2d), and the majority of them (except OE12) exhibited significantly reduced glucose content but no change in xylose content (Figure 2e, f). The results indicated OE‐miR396 leads to reduced lignin and glucose content in switchgrass.

### MiR396 target genes *PvGRF1, 3 and 9* are highly expressed in mature stems

**Figure 3:**
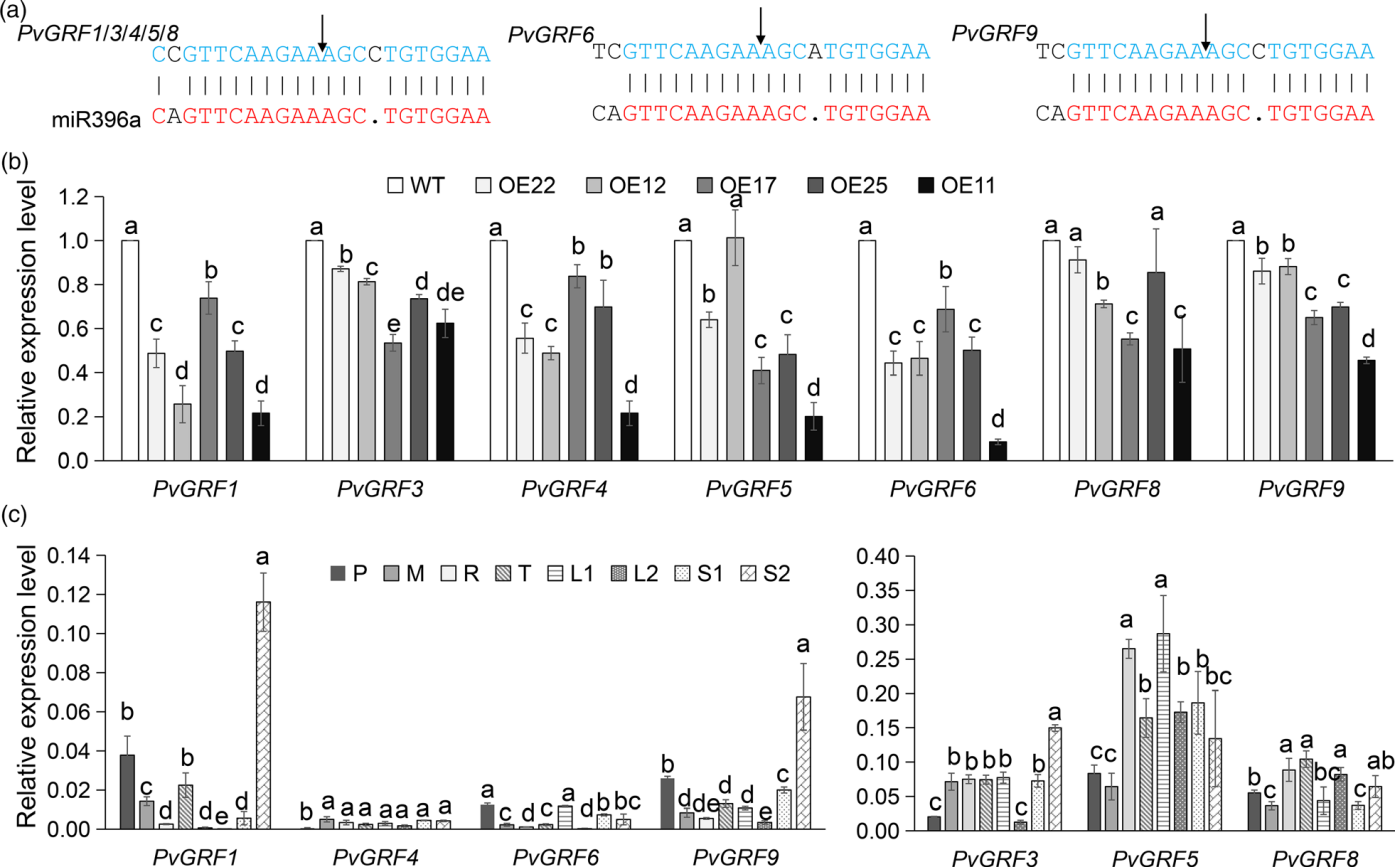
Validation and expression patterns of the miR396 target genes. (a) 5’ RLM‐RACE experimental validation of the miR396a cleavage sites (arrows) of the miR396 putative target genes. (b) The relative expression of the miR396‐target *PvGRFs* in the stem cells (S1) of WT and OE‐miR396 lines revealed by qRT‐PCR analysis (*n* = 3). (c) The expression patterns of the miR396‐target *PvGRFs* in different tissues of switchgrass (*n* = 3). An illustrated photograph of the sampling site is shown in Figure S1b. R, rachilla; M, lemma; P, pistil; T, stamen; stem (S1, S2); leaf (L1, L2); S1 and S2 were sampled from the second internode of the E3 stage tiller; L1 and L2 were sampled 1 cm from the base of the leaves. The error bar indicates standard deviation. Different letters represent significant differences (*P* < 0.05).

MiR396 impacts plant growth and development mainly by post‐transcriptionally repressing the expression of its target *GRF* genes. To reveal the role of GRFs play in miR396‐mediated plant development, we searched the switchgrass genome database using *AtGRFs* and *OsGRFs* as query sequences (Table S2) (Choi et al., 2004) and identified 19 genes with GRF distinctive motifs, WRC and QLQ, and named them based on the phylogenetic analysis results (Figure S4a, b). All the *PvGRF* genes except *PvGRF9* had two homologs distinguished by the letter a and b and were classified into three subfamilies: group Ⅰ (PvGRF1, 2, 3, 4 and 5), group Ⅱ (PvGRF6, 8 and 9) and group Ⅲ (PvGRF10 and 11) (Table S2 and Figure S4a). All, except *PvGRF10a* and *PvGRF11a/b,* had a miR396 binding site in their open reading frames (ORFs) (Table S3). The RNA ligase‐mediated 5’‐rapid amplification of cDNA ends tests (5’ RLM‐RACE) assay revealed that the mRNAs of *PvGRF1*, *3*, *4*, *5*, *6*, *8* and *9* were cleaved by miR396 between base pairs 10 and 11 in the miR396a target site (Figure 3a), and their expression was significantly decreased in most of the OE‐miR396 plants (Figure 3b), further proving that *PvGRF1/3/4/5/6/8/9* were miR396 target genes.

To identify the key *PvGRFs* involved in miR396‐mediated plant height regulation, the expression patterns of the miR396 targeted *PvGRFs* in different tissues of switchgrass were examined. As shown in Figure 3c, *PvGRF1*, *PvGRF9* and *PvGRF3* exhibited significantly higher expression in mature stems than in other tissues. Therefore, we selected *PvGRF1*, *3* and *9* for further analysis.

### *PvGRF1* and *PvGRF9,* but not *PvGRF3,* positively regulate switchgrass plant height and lignin content

To unravel the biological function of the three selected *PvGRFs* in stem development, we generated transgenic switchgrass plants overexpressing *rPvGRFs* (35S:*rPvGRF9*, 35S:*rPvGRF1* and 35S:*rPvGRF3*) and *PvGRFs‐SRDX* (35S: *PvGRF9‐SRDX*, 35S:*PvGRF1‐SRDX* and 35S:*PvGRF3‐SRDX*) that suppress PvGRF1, 3 and 9 activity, respectively (Figure S5a, b).

**Figure 4:**
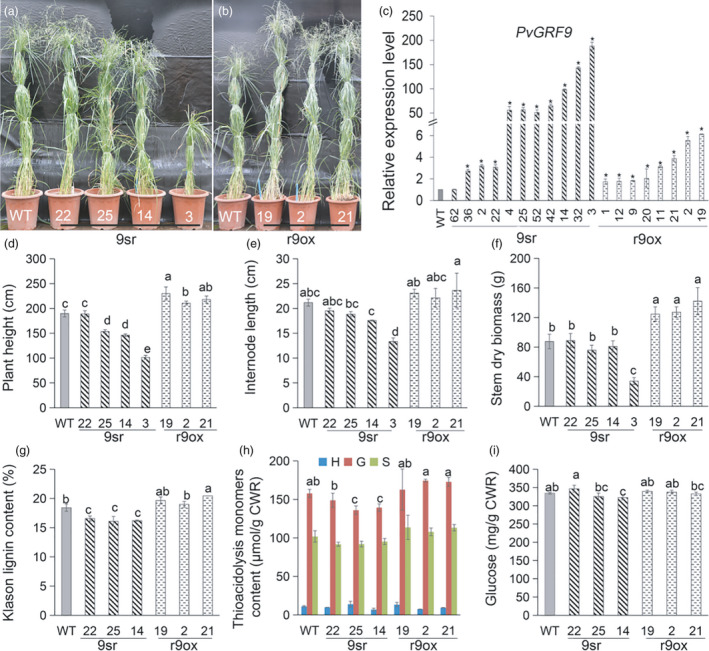
*PvGRF9* positively regulates plant height and lignin content. (a,b) Photograph of typical WT and *PvGRF9‐SRDX* transgenic (9sr) plants (a) and *rPvGRF9* transgenic (r9ox) plants (b). (c) Relative expression of *PvGRF9* in WT, 9sr and r9ox lines (*n* = 3). Asterisk indicates statistically significant differences between WT and TG plants (*P* < 0.05). (d, e) Comparison of the plant height (d), average internode length (e) of WT, 9sr and r9ox lines (*n* = 4). (f) The dry biomass yield of per plant stems of WT, 9sr and r9ox lines (*n* = 4). (g) Comparison of Klason lignin content (*n* = 3) with five technical repeats each. (h) Comparison of lignin monomer content (*n* = 2). (i) Comparison of the glucose yield of the stem cell wall residues of WT, 9sr and r9ox lines (*n* = 3) with five technical repeats. The error bar indicates standard deviation. The different letters indicate statistically significant differences determined by Duncan’s multiple range test (*P* < 0.05).

Seventeen independent transgenic lines overexpressing *rPvGRF9* (r9ox) and twenty *PvGRF9‐SRDX* transgenic (9sr) lines with repression of *PvGRF9* activity were generated (Figure 4a, b) and verified by PCR (Figure S5c) and qRT‐PCR tests (Figure 4c). The 9sr plants showed similar phenotype in plant height as OE‐miR396 plants, and four representative 9sr lines with different *PvGRF9‐SRDX* expression level, 9sr‐22 (about three times above WT), 9sr‐25 and 9sr‐14 (~70 times above WT) and 9sr‐3 (about 200 times above WT) were selected for detailed morphology analysis (Figure 4a). After growing for six months, 9sr‐22 line had similar plant height to WT, 9sr‐25 and 9sr‐14 lines had significantly shorter plant height (about 30 cm) than WT, and 9sr‐3 showed severe dwarf phenotype (shortened by 45% compared to WT) (Figure 4a, d). Further analysis revealed that the shortened plant height of 9sr lines was mainly due to the reduced internode and inflorescence length (Figure 4e; Table S4), which resulted in significantly reduced stem dry biomass yield (Figure 4f). On the contrary, the *rPvGRF9* overexpression switchgrass lines were significantly taller (about 30–50 cm) than the WT controls (Figure 4b, d) due to the elongated inflorescence axis and average internode length (Figure 4e; Table S4). And the stem dry biomass of the r9ox lines was about 1.3 times higher than that of the WT controls (Figure 4e). Additionally, the 9sr lines had slender and shorter leaves than WT, but no significant difference was observed on leaf blade width and length between r9ox lines and WT (Table S4). The results clearly indicate that *PvGRF9* is a positive regulator of switchgrass plant height and biomass yield.

We also generated *rPvGRF1* transgenic plants (r1ox) and *PvGRF1‐SRDX* overexpression transgenic plants (1sr) (Figure S6a–c). The 1sr lines showed a similar phenotype to OE‐miR396 and 9sr lines (Figure S6a, b; Table S5). And plant height, internode length and stem dry biomass showed a significant reduction in the 1sr lines highly expressing *PvGRF1‐SRDX* (Figure S6d–f). The *rPvGRF1* overexpression lines showed significantly improved plant height (about 15% higher than WT) (Figure S6d, Table S5), and a slight improvement, although not significant, in stem dry biomass yield compared to WT controls (Figure S6f). However, transgenic plants overexpressing *rPvGRF3* (r3ox) or *PvGRF3‐SRDX* (3sr) showed no significant difference in plant height from the WT controls (Figure S7a–c). These results indicate *PvGRF1* and *PvGRF9* function redundantly to regulate switchgrass plant height.

We further investigated the effect of *PvGRF1* and *PvGRF9* on stem cell wall composition and observed that repression of *PvGRF9* reduced stem lignin content, mainly due to the reduction of G unit content (Figure 4g, h). On the contrary, r9ox‐21 exhibited significantly increased lignin content compared to WT, the other two *rPvGRF9* overexpression lines, r9ox‐19 and r9ox‐2, also had slightly higher lignin content than the WT controls but had no significant difference (Figure 4g). A positive correlation tendency was also noticed between lignin content and *PvGRF1* expression level (Figure S6g). In addition, repression of PvGRF1 or PvGRF9 activity led to reduced cell wall glucose yield, but had no significant effect on xylose content (Figure 4i; Figure S6h, i).

### Wild‐type *PvGRF9* or *rPvGRF9* restores the impaired phenotype by miR396 overexpression

**Figure 5:**
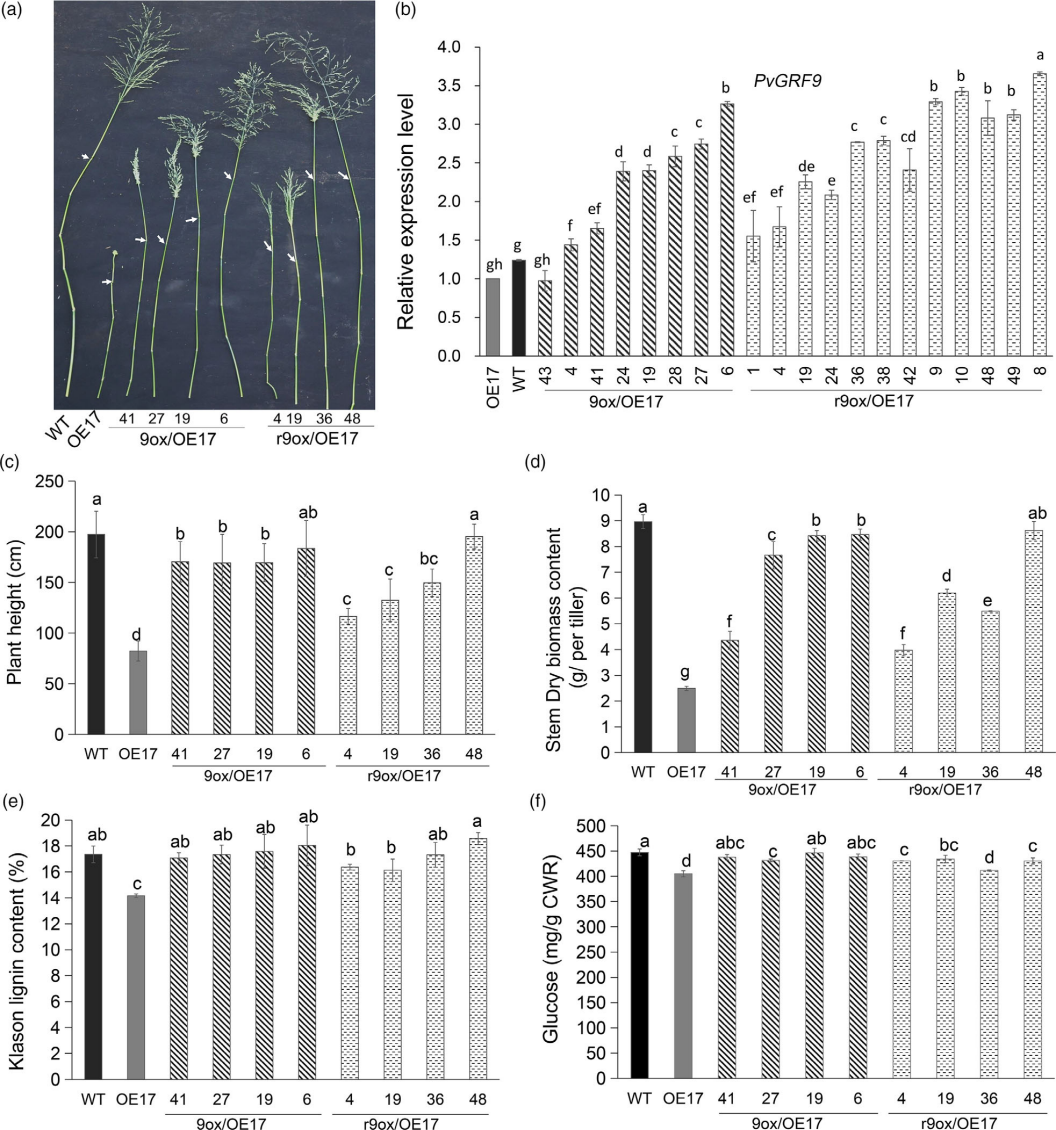
Overexpression of *PvGRF9* or *rPvGRF9* in OE‐miR396 line, OE17 rescued impaired plant phenotype. (a) A photograph of the R3 stage tillers of WT, OE17 and eight representative OE17 plants overexpressing *PvGRF9* (9ox/OE17), or *rPvGRF9* (r9ox/OE17), respectively. The white arrow point to the inflorescence node position. (b) Relative expression of *PvGRF9* in WT, OE17, 9ox/OE17 and r9ox/OE17 by RT‐PCR (*n* = 3). (c) Comparison of plant height of WT, OE17 and (r)9ox/OE17 (*n* = 4) with twenty technical replicates each. (d) The stem dry biomass yield per tiller after culturing six months in greenhouse (*n* = 4). (e, f) Klason lignin content (e) and glucose content (f) of cell wall residues of WT, OE17 and (r)9ox/OE17 (*n* = 3) with five technical repeats each. The error bar indicates standard deviation. The different letters indicate statistically significant differences (*P* < 0.05).

To investigate whether miR396‐mediated suppression of *PvGRFs* caused the aberrant stem height and cell wall lignin content in the OE‐miR396 lines, we conducted functional complementation tests by overexpression of *PvGRF9* and *rPvGRF9* in OE‐miR396 line OE17, respectively (Figure S8a, b; Figure 5a). Intriguingly, depending on the expression level of *PvGRF9* (Figure 5b), the plant height (Figure 5c), stem dry biomass (Figure 5d), leaf width and length and inflorescence morphology of OE17 were restored to WT phenotype in certain degrees (Figure S8c, d; Table S6). The 9ox/OE17‐6 line with the highest *PvGRF9* expression level exhibited a phenotype similar to WT plants (Figure 5a). Similar results were also observed in the OE17 plants overexpressing *rPvGRF9* (r9ox/OE17) (Figure 5a). Further study showed that overexpression of *rPvGRF9* in OE17 significantly increased the length and diameter, but not the number of stem cells (Figure S9a–d). Additionally, the cell wall lignin and glucose content of 9ox/OE17 lines were similar to that of the WT control plants (Figure 5e, f). Although the glucose yield of r9ox/OE17 plants was slightly improved compared to OE17, it remained significantly lower than that of the WT controls (Figure 5f). These results suggest *PvGRF9* and *rPvGRF9* were sufficient to restore the impaired plant height and lignin content in OE‐miR396 transgenic plants.

**Figure 6:**
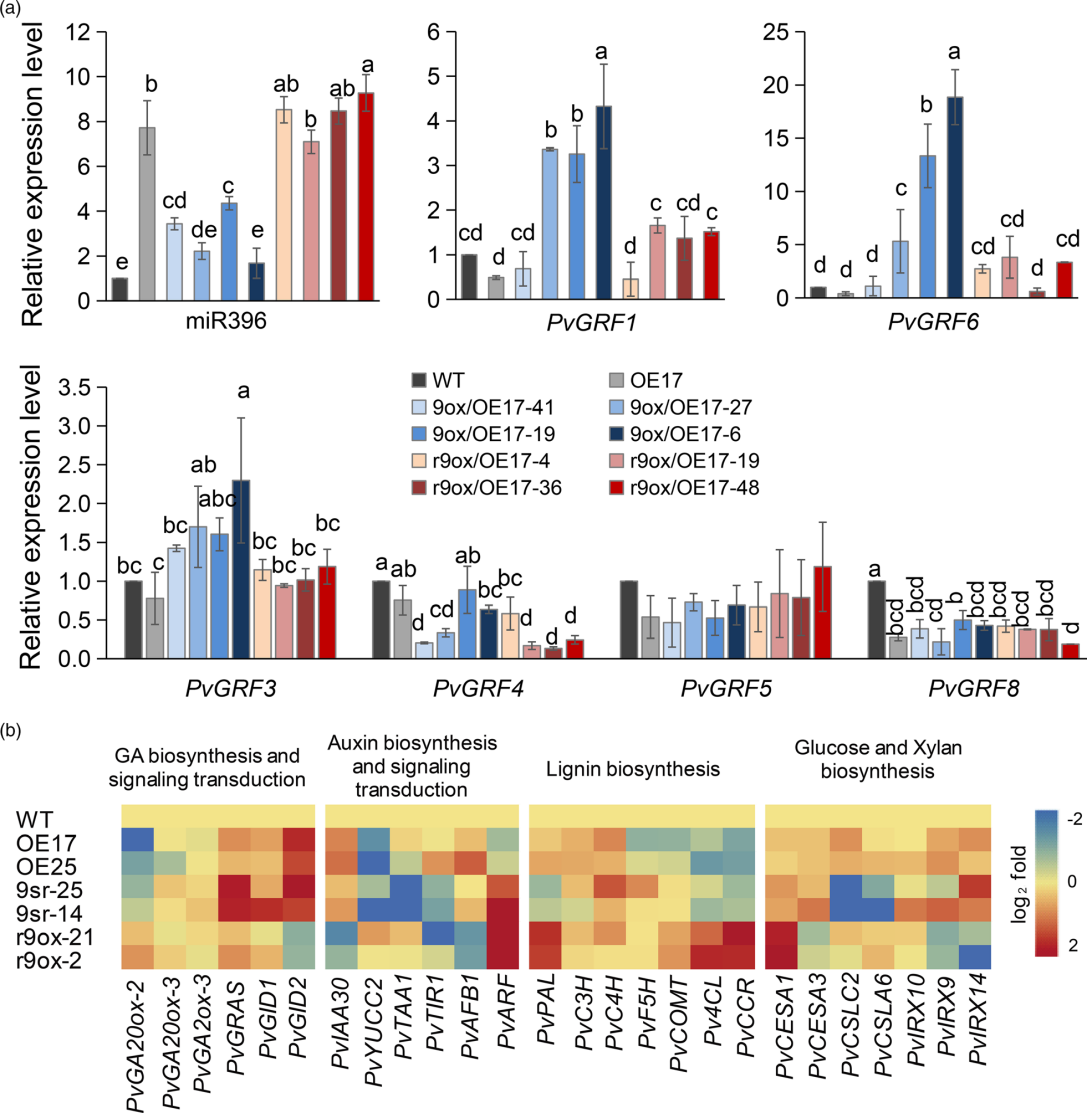
Expression analyses of miR396, miR396‐targeted *PvGRFs* and other related genes in WT and transgenic plants. (a) Relative expression analysis of miR396 and miR396‐targeted *PvGRFs* in WT, OE17 and (r)9ox/OE17 plants (*n* = 3). The error bar indicates standard deviation. The different letters indicate statistically significant differences (*P* < 0.05). (b) Heat map of relative expression levels of the genes in GA and auxin biosynthesis and signalling transduction, lignin biosynthesis, glucose and xylan biosynthesis in WT and transgenic plants. The qRT‐PCR tested data were subjected to log_2_‐fold change (*n* = 2) with three technical repeats for each.

We also tested the expression of miR396 and other *PvGRFs* in 9ox/OE17 and r9ox/OE17 lines to examine the potential effects of *PvGRF9* overexpression on these genes. As expected, the expression level of miR396 of the 9ox/OE17 plants was significantly reduced compared to that of OE17, but still higher than that of WT. However, the expression level of miR396 of the r9ox/OE17 lines was not significantly different from that of OE17 (Figure 6a). Intriguingly, compared to WT and OE17, the expression of *PvGRF1* and *PvGRF6* was significantly increased in 9ox/OE17 lines (except for 9ox/OE17‐41), but was not significantly changed in r9ox/OE17 lines (Figure 6a). However, *PvGRF4* showed a significantly down‐regulated expression in several 9ox/OE17 and r9ox/OE17 lines compared to OE17 (Figure 6a). Clearly, *PvGRF9* impacted the expression of miR396 and its target *PvGRFs* in OE17, which synergistically affected and restored phenotype of OE17.

### Expression analysis of plant development and cell‐wall biosynthesis‐related genes

To explore potential molecular mechanisms of miR396‐*PvGRF9*‐mediated regulation on cell length and cell wall composition, we tested the expression of several genes related to GA and auxin biosynthesis, their signalling transduction and lignin, glucose and xylan biosynthesis by qRT‐PCR (Figure 6b). The results showed that the GA biosynthesis gene *GA20‐oxdiase 2* (*PvGA20ox2*) was significantly down‐regulated in OE‐miR396 plants and *PvGRF9‐SRDX* transgenic plants, while it was significantly up‐regulated in *PvGRF9* overexpressing plants. There were no significant effects on the expression of *PvGA20ox3* and *gibberellin 2‐beta‐dioxygenase 3* (*PvGA2ox‐3*) by miR396‐*PvGRF9* module. The GA signalling transduction‐related genes *GIBBERELLIN INSENSITIVE DWARF1* (*PvGID1*), *PvGID2* and *PvGRAS* showed elevated expression in OE‐miR396 and 9sr lines, while only *PvGID2* was significantly down‐regulated in r9ox lines. *PvYUCC2*, a YUCCA family gene in auxin biosynthesis, was shown to be negatively regulated by miR396‐*PvGRF9* module. *PvTAA1*, a homologous gene of *TRYPTOPHAN AMINOTRANSFERASE OF ARABIDOPSIS 1* in switchgrass, was also significantly down‐regulated in 9sr lines. The auxin signalling transduction and response genes, *auxin signalling F box protein 1* (*PvAFB1*) and *Indole‐3‐acetic acid inducible 30* (*PvIAA30*) showed reduced expression in r9ox lines. *Auxin response factor* (*PvARF*) was up‐regulated in the tested 9sr and r9ox plants. The expression of *4‐coumarate: coenzyme A ligase* (*Pv4CL*) and *cinnamoyl coenzyme A reductase* (*PvCCR*) genes were negatively regulated by miR396‐*PvGRF9* module. *Coumaroyl shikimate 3′‐hydroxylase* (*PvC3H*) and *cinnamate 4‐hydroxylase* (*PvC4H*) genes showed slightly elevated expression in all the tested transgenic plants. *Phenylalanine ammonia‐lyase* (*PvPAL*) gene showed significantly up‐regulated expression in r9ox lines. The tested glucose and xylose biosynthesis genes showed no significant difference between OE‐miR396 lines and WT. *Cellulose synthase‐like A6/C2* (*PvCSLA6/PvCSLC2*) showed significant down‐regulation in 9sr lines. *Irregular xylem 9/14* (*PvIRX9/14*) were also down‐regulated in r9ox lines. Intriguingly, *Cellulose synthase 1* (*PvCESA1*) was up‐regulated in all the tested lines. The results suggest that miR396‐*PvGRF9* module could alter plant height and cell wall composition by, at least partially, disturbing lignin, glucose and xylan biosynthesis‐related genes as well as GA and auxin biosynthesis‐ and transduction‐related genes.

### Overexpression of *rPvGRF9* significantly enhances soluble sugar yield

**Figure 7:**
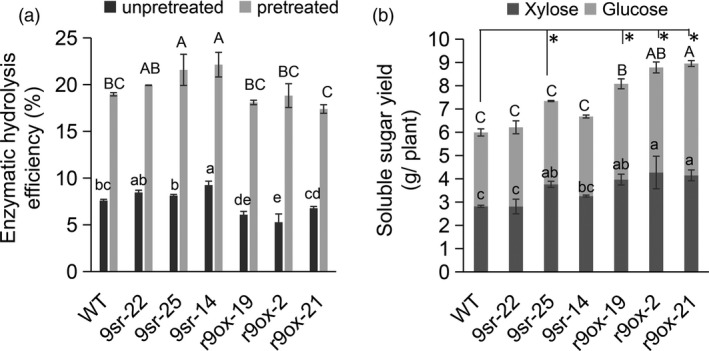
Effects of *PvGRF9‐SRDX* and *rPvGRF9* overexpression on cell wall saccharification. (a) Enzymatic hydrolysis efficiency of cell wall residues of the transgenic plants. (b) The *per* plant contents of glucose and xylan released from WT and transgenic plants after pretreatment. The data are shown as the means of three biological replicates (with five technical repeats each) ± SD. The different letters and asterisks indicate statistically significant differences (*P* < 0.05).

To investigate the practical value of *PvGRF9* in switchgrass genetic modification for biofuel production, we analysed the released glucose and xylose from per gram of CWR with/without pretreatment (Table S7), calculated enzymatic hydrolysis efficiency (EHE) and per plant sugar yield of the WT, 9sr and r9ox plants. The results showed that without pretreatment, r9ox lines released significantly less glucose than WT and 9sr lines. And the xylose yield of r9ox lines was significantly less than that of other tested plants except r9ox‐19, which had no statistical difference from WT and 9sr‐22 (Table S7). After pretreatment, significantly higher glucose, xylose and total soluble sugar yield was also observed in the 9sr lines, 9sr‐14 and 9sr‐25, whereas the glucose and xylose yield of r9ox (except the glucose content of r9ox‐21) showed no significant difference from that of WT controls. Obviously, the r9ox lines tend to reduce EHE, while 9sr lines tend to improve EHE. As shown in Figure 7a, without pretreatment the *PvGRF9* repression line, 9sr‐14, showed significantly elevated EHE, the highest among all the tested lines, while significantly reduced EHE, the lowest of all, was observed in the *rPvGRF9* overexpression line r9ox‐2. After pretreatment, the EHE of the 9sr‐25 and 9sr‐14 lines was significantly higher (about 20%) than that of WT and r9ox lines. Despite statistically insignificant, the EHE of the 9sr‐22 line was also slightly elevated compared to WT controls. Intriguingly, the EHE of the r9ox lines had no significant difference from that of WT after pretreatment (Figure 7a). However, due to the improved biomass yield of the individual r9ox plant, the released per plant glucose yield of the r9ox line was significantly higher than that of the 9sr and WT lines, resulting in significantly higher total soluble sugar yield in the r9ox transgenic plants (about 1.5 times) than that in the WT controls. Sugar yield of the 9sr‐22, 9sr‐14 lines and the WT controls was similar, while that of the 9sr‐25 line had significantly elevated compared to the WT controls (Figure 7b).

## Discussion

Stem of biofuel plants contributes most of its biomass yield and had higher cellulose, hemicellulose and lignin concentration and lower ash contents, which resulted in a better fuel quality than leaves (Tubeileh et al., 2016). MiR396‐*GRF* regulatory pathway has been found to affect the size of various organs in different species (Diao et al., 2018; Gao et al., 2015; Tang et al., 2018). However, the biological function of miR396 and its target genes in regulating plant stem characters for biofuel production has not been demonstrated yet. Moreover, how the expression level of miR396 and *GRF* quantitatively affects growth and development remains unclear (Ercoli et al., 2016; Rodriguez et al., 2010). It is therefore largely unknown how to adjust the balance of miR396 and its target GRF genes *in vivo* to improve feedstock plant biomass yield and characteristics related to biofuel production. Here, we proved that miR396, by suppressing its target *PvGRFs,* especially *PvGRF9,* negatively regulates stem length and stem cell wall lignin content. Our data suggest that up‐regulating the expression of *PvGRF9* may be a promising way to improve switchgrass for biofuel production.

MiR396, one of the evolutionarily conserved miRNAs among angiosperm species, plays multiple roles in plant development (reviewed in Liebsch and Palatnik, 2020; Omidbakhshfard et al., 2015). It was reported that up‐regulating miR396 resulted in a shorter and narrower leaf, dwarf phenotype, abnormal flower and smaller fruit or seed size (Li et al., 2018; Liu et al., 2009; Mecchia et al., 2013; Tang et al., 2018) as well as a severely affected plant growth resulting in a significant reduction in plant biomass (Yuan et al., 2019), which is a negative impact for switchgrass as a biofuel plant. Similarly in this study, overexpression of miR396 reduced leaf size, plant height and panical length in switchgrass. It is well known that miR396 guides *GRF* mRNAs to cleavage or translational arrest to regulate plant growth, and they always play antagonistic roles in regulating plant organs development (Liebsch and Palatnik, 2020; Omidbakhshfard et al., 2015). In *Arabidopsis thaliana*, seven of nine GRFs have miR396 binding sites and post‐transcriptionally repressed by miR396 (Liu et al., 2009; Rodriguez et al., 2010), and all of them involved in leaf development (Debernardi et al., 2014; Horiguchi et al., 2005; Omidbakhshfard et al., 2018; Vercruysse et al., 2020). Herein, we examined nineteen *PvGRFs*, of which thirteen *PvGRFs*’ mRNAs are targeted by miR396. We observed that the expression of miR396‐targeted *GRFs* tend to be repressed significantly in the OE‐miR396 switchgrass plants but with different reduction rates, which might lead to morphology differences of the OE‐miR396 lines. *PvGRF5* showed reduced expression in miR396‐overexpressing lines except OE12; meanwhile, a slight difference in some of the cell wall compositions between OE12 and other transgenic lines was also noticed. It was reported that the function of *AtGRF5* (belonging to the same subgroup as *PvGRF5*) in improving leaf size cannot be replaced by other family members (Horiguchi et al., 2005). Arguably, the difference in some of the cell wall compositions between OE12 and other transgenic lines could be attributable to their differential *PvGRF5* suppression, which might result from the position effect for transgene insertion that could differentially impact other mechanisms than miR396 involved in *PvGRF5* regulation in different transgenic lines. It should be noted that the expression of miR396 was not tightly associated with how much its targeted *PvGRFs* were repressed in the tissues analysed, further suggesting a possible complicated relationship between miR396 and *PvGRFs* in switchgrass. Similarly, a loose negative effect of miR319 on the expression of its targeted genes in different tissues has previously been reported in switchgrass and rice (Liu et al., 2020; Yang et al., 2013).

Function of *GRF* genes is partially overlapped, but individual *GRF* also has its specific function in regulating plant growth and development (Omidbakhshfard et al., 2015). Repressing miR396 activity to indiscriminately up‐regulate *GRFs’* expression always results in deleterious effects (Liang et al., 2014; Liu et al., 2014). Therefore, characterization and utilization of individual *GRF* genes with specific functions have been recognized as highly promising strategies for modifying specific traits (Li et al., 2018; Luo et al., 2005; Rossmann et al., 2020). Overexpression of *OsGRF4* or mutation of two bases in miR396 target site of *OsGRF4* were sufficient to increase rice yield without a significant impact on other traits (Duan et al.,^2015^; Li et al., 2018). In this study, we focused on characterizing the *PvGRFs* with specific functions in switchgrass plant height, which is related to stem elongation. It was reported that *OsGRF1* has been reported to repress stem elongation when ectopically expressed in *Arabidopsis* but have no effect on leaf size (Van Der Knaap et al., 2000). However, *ZmGRF1* negatively regulated plant height but enhanced leaf size (Nelissen et al., 2015). Our data clearly indicated that overexpression of *rPvGRF1* significantly improved switchgrass plant height, but had no obvious effect on leaf size, suggesting the *GRF* homologs may have different functions in different plant species. The functional differentiation of the *GRF* homologs has also been reported in *OsGRF10* and *ZmGRF10* (both of them lack miR396‐binding site) for their impact on plant height (Kuijt et al., 2014; Wu et al., 2014). *AtGRF9* belongs to the same evolutionary subclass as *GRF10* and reportedly plays a negative role in regulating organism size (Omidbakhshfard et al., 2018). In this study, we found *PvGRF9* (homologous gene of *AtGRF1* and *AtGRF2* in switchgrass) had functional redundancy with *PvGRF1,* but exhibited stronger impact in regulating switchgrass plant height than *PvGRF1*. Overexpression of *PvGRF9* or *rPvGRF9* in OE‐miR396 background was sufficient to rescue impaired plant height, indicating *PvGRF9* plays a dominant role in regulating switchgrass stem elongation. Overexpression of *PvGRF9* also reduced the expression of miR396 in OE17 and increased the expression of *PvGRF1*, *PvGRF3* and *PvGRF6*, of which *PvGRF3* was verified to have no significant effect on plant height. In rice, *OsGRF6* has been reported to positively regulate plant height (Tang et al., 2018). Therefore, in addition to *PvGRF1* and *PvGRF9*, *PvGRF6* might also play a positive role in regulating switchgrass plant height, which needs to be further studied.

MiR396‐*GRF* network is also associated with cell proliferation of organs (Tang et al., 2018; Omidbakhshfard et al., 2018). In this study, we found *rPvGRF9* rescued the stem cell length and diameter of OE‐miR396 plant, but not cell number. The alteration in cell size is often accompanied by the change in plant cell wall components (Fu et al., 2012; Voorend et al., 2016; Wuddineh et al., 2015). We found miR396‐*GRF* module negatively affected cell wall lignin content in switchgrass by altering the G‐lignin monomer content. GRFs were reported to interact with plant hormones such as GA, brassinosteroid (BR) and auxin (Gao et al., 2015; Tang et al., 2018). It is well known that GA is one of the main hormones affecting plant growth and development (Tong et al., 2014; Voorend et al., 2016; Wuddineh et al., 2015). Elevating GA level by overexpression of *GA20ox* increased plant height and lignin content (Do et al., 2016; Voorend et al., 2016). Reducing endogenous GA level, in switchgrass, by overexpression of *GA2ox5/9* gene resulted in a dwarf plant phenotype (Wuddineh et al., 2015). In rice, reducing *OsGRF6* expression level reportedly hindered GA biosynthesis and signalling transduction, which contributed to a reduced plant height (Tang et al., 2018). Here, we found miR396‐*PvGRF9* module negatively regulated expression of *GA20ox‐2* gene, which could contribute to the alteration in plant height and cell wall composition. However, different response patterns of the GA signalling transduction‐related genes to miR396 have been observed between switchgrass and rice, especially for *PvGID2* (Tang et al., 2018). GID2 is a subunit of Skp1‐Cullin‐F box protein (SCF) E3 ubiquitin ligase and involved in the degradation of DELLA proteins, a GAs signalling hub (Ueguchi‐Tanaka et al., 2008; Van De Velde et al., 2017). Recently, it was reported that the only DELLA protein in rice, SLENDER RICE1 (SLR1), regulates miR396 expression to control cell proliferation (Lu et al., 2020). These results suggest a complex regulatory pattern may exist to regulate GA signalling transduction by miR396 in switchgrass. *OsGRF6* has previously been reported to positively regulate auxin biosynthesis and signalling pathway by directly activating the expression of *OsYUCCA1* and *OsARF11* (Gao et al., 2015). In this study, we also found *PvYUCC2* was negatively regulated by miR396‐*PvGRF9* module in switchgrass. However, *PvARF* was highly expressed in all the tested 9sr and r9ox lines. These results suggest miR396‐*PvGRF9* module may play a conserved role in negatively regulating auxin biosynthesis in switchgrass and rice. MiR396‐*PvGRF9* module negatively regulated lignin biosynthesis genes *Pv4CL* and *PvCCR* and disturbed other lignin biosynthesis genes examined, such as *PvC3H* and *PvC4H*. It has been reported that down‐regulation of *4CL* or *CCR* gene expression in different plant species decreased the plant lignin content and improved the cellulose saccharification, resulting in higher sugar yield (Chabannes et al., 2001; Kawasaki et al., 2006; Park et al., 2017; Van Acker et al., 2014). Although lower glucose content was observed in most OE‐miR396 lines than controls, the expression of the glucose biosynthesis genes tested was not significantly impacted by miR396 overexpression. However, the *PvCSLA2* and *PvCSLC6* were significantly down‐regulated in the 9sr lines, which may attribute to the reduced glucose content (Goubet et al., 2009). We also found the xylan biosynthesis genes, *PvIRX9* and *PvIRX14* were down‐regulated by r9ox (Hu et al., 2016). These results indicate miR396‐*PvGRF9* disturbed GA and auxin biosynthesis and signalling transduction and the expression of lignin, glucose and xylan biosynthesis‐related genes to modulate, at least partially, the plant height and cell wall composition. The detailed molecular regulatory pathway remains to be further investigated.

The content and composition of lignin (especially G‐lignin content) play important roles in impeding the effect of polysaccharides hydrolysis to produce sugar (Bhatia et al., 2017; Li et al., 2014; Pei et al., 2016). In this study, the lignin content and biomass yield were both increased by overexpression of *PvGRF9*, which also resulted in significantly reduced sugar releasing efficiency in the r9ox lines. On the contrary, the sugar releasing efficiency in the 9sr lines tend to be improved with the 9sr‐14 line having the highest among all the tested plants, and the strongest repression of *PvGRF9* was also observed in 9sr‐14 line. The enzymatic hydrolysis efficiency of cell wall (without pretreatment) of *PvGRF9* transgenic plants had an obvious negative correlation with its lignin content and G‐lignin content. However, after pretreatment, the enzymatic hydrolysis efficiency of the r9ox plants had no significant difference from that of the WT controls, indicating that pretreatment and hydrolysis condition offset the inhibitory effect caused by the increase of lignin content in the r9ox plants (Financie et al., 2016; Zhang and Wu, 2014; Zhang et al., 2017). Due to the significant increase in biomass yield and the similar enzymatic hydrolysis efficiency after pretreatment, overexpression of *rPvGRF9* in switchgrass would be beneficial for biofuel production.

In conclusion, we have provided evidence demonstrating miR396 negatively affects the size of leaf, inflorescence and stem growth in switchgrass. Among the thirteen miR396‐targeted *PvGRFs*, *PvGRF1* and *PvGRF9* play redundant roles involved in miR396‐mediated regulation of the stem growth. MiR396‐*PvGRF* module negatively regulates switchgrass biomass, cell wall lignin and glucose yield. Up‐regulating *rPvGRF9* switchgrass lines resulted in an ideal plant phenotype with significantly higher biomass yield and per plant sugar release. Overexpression of *PvGRF9* could be used as a valuable molecular tool for switchgrass plant height and biomass yield improvement for biofuel production.

## Materials and methods

### Plant materials and growth conditions

Switchgrass (*Panicum virgatum* L.) cultivar Alamo was used in the experiments. Wild‐type and transgenic plants were cultured in a greenhouse with a photoperiod of 14 h/10 h (day/night) for four months and then clonally propagated from tillers, four individuals of each line were used as biological replicates. After six months of growth, at least twenty R3 stage tillers (fully emerged spikelets and peduncle) (Hardin et al., 2013) of each plant (as technical replicates) were used to collect data of morphology indexes. And then, twenty R3 stage tillers per switchgrass plant (as one biological replicate) were harvested, weighed and oven‐baked at 65 °C for 48 h. Upon removal of the leaves and inflorescences, the stems were ground and filtered by 0.6 mm screen as dried‐well materials of stems (DMS).

### Plasmid construction and plant transformation

For overexpression of miR396, full‐length cDNA of O*sa‐miR396a* (GQ419538) was cloned into the *Xba* Ⅰ and *Sal* Ⅰ restriction enzyme cutting sites of a binary vector pZh01 (Xiao et al., 2003). For overexpression of *PvGRFs*, the full‐length cDNA of *PvGRF1* (Pavir.1NG528000), *PvGRF3* (Pavir.7KG300700) and *PvGRF9* (Pavir.9NG094400) were cloned and used as templates for bridging PCR to produce the ORFs of the *rPvGRFs,* in which synonymous mutations abolished the miR396 target sites. The individual *rPvGRFs* were subcloned into pZh01, respectively. To repress the function of PvGRFs, the 3’ ends of *PvGRFs* were linked to a sequence encoding the SRDX domain and cloned into pZh01 (Liu et al., 2020; Wu et al., 2016). All of these genes were under the control of the CaMV 35S promoter. The *hpt* gene was used as the marker gene, and hygromycin B was used as a selection reagent in producing transgenic plants. The T‐DNA region of the expression vectors used for transformation was shown in Figures S2a and S5a. The primers used in this study are listed in Table S8.

Callus induced by one Alamo seed was used for *Agrobacterium*‐mediated transformation as we previously reported (Liu et al., 2015). The calli induced from the young inflorescence of the R1 stage tillers of the selected miR396 overexpression line, OE17, were subjected to transformation with pZH01‐35S:*PvGRF9* and pZH01‐35S:*rPvGRF9* constructs, respectively (Liu et al., 2015).

### MiR396 cleavage site analysis

The 5’ RLM‐RACE were used to detect the miR396 cleavage site in the predicated target genes following Wang and Fang (2015). At least twenty clones of each gene were picked and sequenced. The primers and 5’ adaptor sequences were listed in Table S8.

### Isolation and analysis of plant DNA and RNA

Genomic DNA was extracted from leaves of switchgrass plants using the CTAB method for PCR tests (Liu et al., 2015). Total RNA was isolated using TRIzol reagent (Liu et al., 2017). Following the manufacturer’s instruction, 1 μg of RNA was reverse transcribed to synthesize the first strand cDNA using the protocol of a Primer Script RT reagent kit (Takara, Dalian, China RR047A) with the DNA eraser function. The random primer oligo (dT) or stem‐loop RT primer (for miR396) were used to synthesize first strand cDNA in reverse transcription reaction (Liu et al., 2017). The cDNA was used as a template for qRT‐PCR analysis using SYBR Green supermix (Takara, Dalian, China RR420). The primers used in the experiments were listed in Table S8. All data were collected following EcoTM Real‐Time PCR System User Guide (Illumina, Westlake Village, CA, USA, EC‐100‐1001, CA). Analysis of the relative gene expression was carried out by the change in Ct method using a ubiquitin gene (AP13CTG25905) or the nuclear small RNA U6 cDNA (Pavir.J34795.1) (for miR396) of switchgrass as internal control (Liu et al., 2017). The data for relative gene expression levels were means derived from at least three biological replicates. Specifically, log_2_‐fold changes of means derived from two biological replicates and three technical repeats were presented in heat map generated with Excel 2010 and Adobe Illustrator software (CC 2017).

### Histological and scanning electron microscopy analyses

To visualize lignin content in switchgrass stem, switchgrass hand‐cut cross sections of the middle of 1NE3 stem were stained with 1% phloroglucinol/HCl for 1 min. Digital images were captured under a light microscope (Nikon, Shizuoka, Japan C‐DSS230) (Liu et al., 2020). The middle parts of 1NE3 stems were fixed in 2.5% glutaraldehyde. Stem epidermal cells were photographed under scanning electron microscope (SEM). The cell diameter of cross section cells (*n* = 80) and long cell length of epidermal cells (*n* = 50) were measured from SEM photographs using ImageJ software (https://imagej.en.softonic.com). The cell number was calculated by the ratio of the internode length to the long cell length (*n* = 50).

### Determination of acetyl bromide (AcBr) lignin content

CWR (20.5 mg) was weighed into a reaction bottle with 5 mL acetyl bromide reagent (25%, V/V) and heated to 50 °C for 4 h. The supernatant (4 mL) was then transferred into a 50‐mL volumetric flask, to which 10 mL of 2 m NaOH, 12 mL of acetic acid and 1 mL of 0.5 m hydroxylamine were added followed by the addition of glacial acetic acid to bring the total volume to 50 mL. The absorbance of the mixture at 280 nm was then measured by a spectrophotometer (Fu et al., 2012).

### Lignin monomer content determination

The lignin monomers were identified and quantified by GC‐MS analysis using gas chromatograph (Hewlett‐Packard 5890 series II) with a series of mass selective detector (5971) according to the thioacidolysis method (Lapierre et al., 1995; Liu et al., 2020). Two biological duplicates of each line were used for analysis.

### Determination of Klason lignin content and carbohydrate yield

DMS samples were sequentially washed with chloroform:methanol (2:1, V:V), methanol, 50% methanol, and Mili‐Q water. Each step took 30 min and the whole process was repeated three times. The residues were dried in vacuum machine as dried‐well cell wall residue (CWR) (Fu et al., 2011; Liu et al., 2020). A two‐stage acid hydrolysis method was used for determining Klason lignin content and carbohydrate yield as we previously reported (Liu et al., 2020). All the presented data were mean from three biological replicates (with five technical replicates each) of each line.

### Cell wall enzymatic hydrolysis analysis

For enzymatic hydrolysis efficiency determination, CWR was pretreated with 10 g/L NaOH or ultrapure water (as unpretreated) and then exposed to a cellulase and cellobiase mixture (Imperial Jade Biotechnology Ningxia, China Co., Ltd) for 72 h incubating at 50℃ (Liu et al., 2020). The supernatant was used to detect the monomeric sugar content (Peng et al., 2015). The monomeric sugars (glucose, xylose) were determined by HPLC system equipped with a Hi‐Plex Ca column (7.7 × 300 mm, Agilent Technology, USA), LC‐20AT pump (Shimadzu, Japan) and RID‐10A refractive index detector (Shimadzu, Japan) (Peng et al., 2015). The enzymatic hydrolysis efficiency (%) was determined by the ratio of the summation of glucose and xylose released by enzymatic hydrolysis to the amount of sugar (glucose, xylose) present in the cells wall composition. The solubility sugar yield = the per plant biomass (g) × CWR content × cell wall carbohydrate yield (g/g CWR) × enzymatic hydrolysis efficiency (Liu et al., 2020). Three biological replicates (with five technical repeats each) were used in the experiments.

### Phylogenetic analysis

The full‐length amino acid sequences used in this study were obtained from database Phytozome (www.phytozome.net) or NCBI (www.ncbi.nlm.nih.gov) (the accession numbers were listed in Table S2). These sequences were used to create alignments with the Clustal W. The phylogenetic tree was generated by the MEGA 5.0 (https://www.megasoftware.net/) program. Comparison of the amino acid sequences was performed by the multiple sequence alignment progress of DNAMAN8.0. (Lynnon Biosoft, San Ramon, CA, USA)

### Statistical analysis

The one‐way analysis of variance (ANOVA) was used for data analysis. The comparison of treatments was separated by Duncan’s multiple range test (*P* < 0.05). The proc GLM for ANOVA of SAS 8.2 (SAS Institute, Cary, NC) was used for the analyses.

## Conflicts of interest

The authors declare that they have no conflict of interests.

## Author Contributions

Y.R.L and W.J.Z conceived and designed the experiments; Y.R.L, J.P.Y, W.K.X and R.Y performed the experiments; Y.R.L, D.Y.L and H.L analysed the data; Y.R.L, H.L and W.J.Z wrote and revised the manuscript. All authors read and approved the final version of the manuscript.

## Supporting information

**Table S1** Statistical analysis of morphological traits of WT and OE‐miR396 plants
**Table S2** List of the Genebank accession numbers of the sequences used in this study
**Table S3** Target site analyses of the putative miR396 targeted *PvGRFs*
**Table S4** Statistical analyses of the morphological parameters of the WT and transgenic plants overexpressing *PvGRF9‐SRDX* (9sr) and *rPvGRF9* (r9ox)
**Table S5** Statistical analysis of the morphological parameters of the WT and transgenic plants overexpressing *PvGRF1‐SRDX* (1sr) and *rPvGRF1* (r1ox)
**Table S6** The morphological characteristics of the transgenic plants
**Table S7** Cell wall enzymatic hydrolysis analysis of WT and transgenic plants overexpressing *PvGRF9‐SRDX* (9sr) and *rPvGRF9* (r9ox)
**Table S8** A list of primers used in this study
**Figure S1** The sequence alignment and expression pattern of miR396
**Figure S2** Production of the *Osa‐MIR396a* transgenic switchgrass plants
**Figure S3** Scanning electron microscopy of the middle part of the first internode (top) cross section and epidermal cells of the E3 stage tiller (the first internode from the top, 1NE3)
**Figure S4** Sequence alignment of the GRF proteins of switchgrass, rice and *Arabidopsis*
**Figure S5** The schematic map of the *PvGRFs*‐related gene constructions and PCR analysis of *PvGRF9*‐related genes in transgenic plants
**Figure S6***PvGRF1* positively regulates plant height and lignin content
**Figure S7***PvGRF3* showed no significant effect on switchgrass plant height
**Figure S8** The example of transgene insertion revealed by PCR analysis and the morphological characteristics of the wild type (WT) and complementation OE17 plants
**Figure S9** Scanning electron microscopy of the middle part of the first internode (top) cross section and epidermal cells, leaf and leaf sheath of the E3 stage tiller (the first internode from the top, 1NE3)
