# Supplementary material for: MiR396‐GRF module associates with switchgrass biomass yield and feedstock quality
Source: Plant Biotechnol J. 2021 Feb 24;19(8):1523–36. doi: 10.1111/pbi.13567 (PMC8384596; doi:10.1111/pbi.13567)
Supplement: Supplementary file 1 — Table S1 Statistical analysis of morphological traits of WT and OE‐miR396 plants Table S2 List of the Genebank accession numbers of the sequences used in this study Table S3 Target site analyses of the putative miR396 targeted PvGRFs Table S4 Statistical analyses of the morphological parameters of the WT and transgenic plants overexpressing PvGRF9‐SRDX (9sr) and rPvGRF9 (r9ox) Table S5 Statistical analysis of the morphological parameters of the WT and transgenic plants overexpressing PvGRF1‐SRDX (1sr) and rPvGRF1 (r1ox) Table S6 The morphological characteristics of the transgenic plants Table S7 Cell wall enzymatic hydrolysis analysis of WT and transgenic plants overexpressing PvGRF9‐SRDX (9sr) and rPvGRF9 (r9ox) Table S8 A list of primers used in this study Figure S1 The sequence alignment and expression pattern of miR396 Figure S2 Production of the Osa‐MIR396a transgenic switchgrass plants Figure S3 Scanning electron microscopy of the middle part of the first internode (top) cross section and epidermal cells of the E3 stage tiller (the first internode from the top, 1NE3) Figure S4 Sequence alignment of the GRF proteins of switchgrass, rice and Arabidopsis Figure S5 The schematic map of the PvGRFs‐related gene constructions and PCR analysis of PvGRF9‐related genes in transgenic plants Figure S6 PvGRF1 positively regulates plant height and lignin content Figure S7 PvGRF3 showed no significant effect on switchgrass plant height Figure S8 The example of transgene insertion revealed by PCR analysis and the morphological characteristics of the wild type (WT) and complementation OE17 plants Figure S9 Scanning electron microscopy of the middle part of the first internode (top) cross section and epidermal cells, leaf and leaf sheath of the E3 stage tiller (the first internode from the top, 1NE3) [file PBI-19-1523-s001.docx]

**Supplementary data**

**Table S1.** Statistical analysis of morphological traits of WT and OE-miR396 plants.

| Line | Rachis length^&^ | Tiller number^￥^ | Leaf blade length^*^ | Leaf blade width^*^ |
| --- | --- | --- | --- | --- |
| WT | 94.32±4.07a | 25.50±5.97a | 62.03±4.18a | 16.73±0.88a |
| OE11 | -- | 20.50±3.87a | 21.54±2.80d | 5.86±0.38d |
| OE17 | 42.33±9.35b | 24.50±3.70a | 50.09±8.27b | 15.12±0.72b |
| OE25 | 41.80±10.35b | 26.25±6.40a | 49.19±8.23b | 15.10±0.76b |
| OE12 | 90.58±2.06a | 23.75±4.11a | 63.39±6.87a | 16.47±1.17a |
| OE22 | 87.73±7.90a | 25.75±9.22a | 61.57±5.79a | 16.32±1.28a |

^&^ Rachis length was measured from the R3 stage tillers. ^*^ The leaf length and width of the second leaves of the R3 stage tillers from top were measured. The leaf blade width was measured at the position 1 cm from the base of leaves. Data are shown as the means of four biological replicates (with 20 technical repeats each) ± SD (n=4). Different letters in the same column represent significant differences (*P* < 0.05). ^￥^Tiller number per plant. Data are shown as the means ± SD (n=4).

**Table S2.** List of the GenBank accession numbers of the sequences used in this study.

| **Gene name** | **Locus name/**  **accession number** | **Species** | **Reference** |
| --- | --- | --- | --- |
| PvGRF1a | Pavir.J03212 | *Panicum virgatum* | Fig.S4 |
| PvGRF1b | Pavir.J14128 | *P. virgatum* | Fig.S4 |
| PvGRF2a | Pavir.Da01847 | *P. virgatum* | Fig.S4 |
| PvGRF2b | Pavir.4NG128900 | *P. virgatum* | Fig.S4 |
| PvGRF3a | Pavir.Ga00724 | *P. virgatum* | Fig.S4 |
| PvGRF3b | Pavir.Gb00674 | *P. virgatum* | Fig.S4 |
| PvGRF4a | Pavir.Aa00789 | *P. virgatum* | Fig.S4 |
| PvGRF4b | Pavir.Ab02786 | *P. virgatum* | Fig.S4 |
| PvGRF5a | Pavir.Da02366 | *P. virgatum* | Fig.S4 |
| PvGRF5b | Pavir.Db02421 | *P. virgatum* | Fig.S4 |
| PvGRF6a | Pavir.J11763 | *P. virgatum* | Fig.S4 |
| PvGRF6b | Pavir.J29705 | *P. virgatum* | Fig.S4 |
| PvGRF8a | Pavir.Ha01401 | *P. virgatum* | Fig.S4 |
| PvGRF8b | Pavir.Hb00712 | *P. virgatum* | Fig.S4 |
| PvGRF9a | Pavir.Ia01043 | *P. virgatum* | Fig.S4 |
| PvGRF10a | Pavir.Aa00919 | *P. virgatum* | Fig.S4 |
| PvGRF10b | Pavir.J06517 | *P. virgatum* | Fig.S4 |
| PvGRF11a | Pavir.Ba01449 | *P. virgatum* | Fig.S4 |
| PvGRF11b | Pavir.Bb02610 | *P. virgatum* | Fig.S4 |
| AtGRF1 | AT2G22840 | *Arabidopsis thaliana* | Choi et al., 2004 |
| AtGRF2 | AT4G37740 | *A. thaliana* | Choi et al., 2004 |
| AtGRF3 | AT2G36400 | *A. thaliana* | Choi et al., 2004 |
| AtGRF4 | AT3G52910 | *A. thaliana* | Choi et al., 2004 |
| AtGRF5 | AT3G13960 | *A. thaliana* | Choi et al., 2004 |
| AtGRF6 | AT2G06200 | *A. thaliana* | Choi et al., 2004 |
| AtGRF7 | AT5G53660 | *A. thaliana* | Choi et al., 2004 |
| AtGRF8 | AT4G24150 | *A. thaliana* | Choi et al., 2004 |
| AtGRF9 | AT2G45480 | *A. thaliana* | Choi et al., 2004 |

**Table S2.** Continued.

| **Gene name** | **Locus name/**  **accession number** | **Species** | **Reference** |
| --- | --- | --- | --- |
| OsGRF1 | LOC_Os02g53690 | *Oryza sativa* ssp*. Japonica* | Choi et al., 2004 |
| OsGRF2 | LOC_Os06g10310 | *O. sativa* ssp*. J.* | Choi et al., 2004 |
| OsGRF3 | LOC_Os04g51190 | *O. sativa* ssp*. J.* | Choi et al., 2004 |
| OsGRF4 | LOC_Os02g47280 | *O. sativa* ssp*. J.* | Choi et al., 2004 |
| OsGRF5 | LOC_Os06g02560 | *O. sativa* ssp*. J.* | Choi et al., 2004 |
| OsGRF6 | LOC_Os03g51970 | *O. sativa* ssp*. J.* | Choi et al., 2004 |
| OsGRF7 | LOC_Os12g29980 | *O. sativa* ssp*. J.* | Choi et al., 2004 |
| OsGRF8 | LOC_Os11g35030 | *O. sativa* ssp*. J.* | Choi et al., 2004 |
| OsGRF9 | LOC_Os03g47140 | *O. sativa* ssp*. J.* | Choi et al., 2004 |
| OsGRF10 | LOC_Os02g45570 | *O. sativa* ssp*. J.* | Choi et al., 2004 |
| OsGRF11 | LOC_Os07g28430 | *O. sativa* ssp*. J.* | Choi et al., 2004 |
| OsGRF12 | LOC_Os04g48510 | *O. sativa* ssp*. J.* | Choi et al., 2004 |
| PvC4H | AP13CTG28733 | *P. virgatum* | - |
| PvCOMT | Pavir.J35577 | *P. virgatum* | - |
| Pv4CL | AP13CTG06049 | *P. virgatum* | - |
| PvCCR | Pavir.Fa00937 | *P. virgatum* | - |
| PvPAL | Pavir.Ab02345 | *P. virgatum* | - |
| PvC3H | AP13ISTG41630 | *P. virgatum* | - |
| PvF5H | Pavir.Ia01427 | *P. virgatum* | - |
| PvGA20ox-2 | Pavir.J11513.1 | *P. virgatum* | - |
| PvGA20ox-3 | Pavir.Ib04505.1 | *P. virgatum* | - |
| PvGA2ox-3 | Pavir.Eb03210.1 | *P. virgatum* | - |
| PvGRAS | Pavir.2NG525400.1 | *P. virgatum* | - |
| PvGID1 | Pavir.J583000.1 | *P. virgatum* | - |
| PvGID2 | Pavir.1NG310200.1 | *P. virgatum* | - |
| PvCESA1 | AP13CTG06092 | *P. virgatum* | Wuddineh et al., 2016 |
| PvCESA3 | AP13CTG00607 | *P. virgatum* | Wuddineh et al., 2016 |
| PvCSLC2 | AP13CTG06284 | *P. virgatum* | Wuddineh et al., 2016 |
| PvCSLA6 | AP13CTG14018 | *P. virgatum* | Wuddineh et al., 2016 |
| PvIRX10 | Pavir.Eb03725 | *P. virgatum* | - |
| PvIRX9 | Pavir.J35635 | *P. virgatum* | - |
| PvIRX14 | Pavir.Da00118 | *P. virgatum* | - |
| PvIAA30 | Pavir.9KG079100 | *P. virgatum* | - |
| PvYUCC2 | Pavir.5KG529600 | *P. virgatum* | - |
| PvTAA1 | Pavir.5KG073100 | *P. virgatum* | - |
| PvTIR1 | Pavir.J05101.1 | *P. virgatum* | - |
| PvAFB1 | Pavir.J08245.1 | *P. virgatum* | - |
| PvARF | Pavir.3KG402700 | *P. virgatum* | - |

**Table S3.** Target site analyses of the putative miR396 targeted *PvGRFs.*

| Gene name | miR396a / miR396 target site |
| --- | --- |
| miR396a: | gucaaguucuuucgacaccuu |
| PvGRF1a | ccguucaagaaagccugugga |
| PvGRF1b | ccguucaagaaagccugugga |
| PvGRF2a | ccguucaagaaagccugugga |
| PvGRF2b | ccguucaagaaagccugugga |
| PvGRF3a | ccguucaagaaagccugugga |
| PvGRF3b | ccguucaagaaagccugugga |
| PvGRF4a | ccguucaagaaagccugugga |
| PvGRF4b | ccguucaagaaagccugugga |
| PvGRF5a | ccguucaagaaagccugugga |
| PvGRF5b | ccguucaagaaagccugugga |
| PvGRF6a | guucaagaaagcauguggaa |
| PvGRF6b | guucaagaaagcauguggaa |
| PvGRF8a | ccguucaagaaagcaugugga |
| PvGRF8b | ccguucaagaaagcaugugga |
| PvGRF9a | guucaagaaagccuguggaa |
| PvGRF10a | - |
| PvGRF10b | ccguucaagaaagccugugga |
| PvGRF11a | - |
| PvGRF11b | - |

“-” represents non-target site predicted by miRBase.

**Table S4.** Statistical analyses of the morphological parameters of the WT and transgenic plants overexpressing *PvGRF9-SRDX* (9sr) and *rPvGRF9* (r9ox).

| Lines | Stem diameter (mm) ^§^ | Leaf length (cm) ^*^ | Blade width (mm) ^*^ | Inflorescence length (cm) ^&^ | Internode number^&^ |
| --- | --- | --- | --- | --- | --- |
| WT | 4.06 ± 0.55 abc | 62.68± 0.75 a | 13.72± 0.39 ab | 77.86± 1.27 ab | 4~6 |
| 9sr-22 | 3.72± 0.03 bc | 60.51± 1.40 ab | 12.21± 0.11 b | 65.08± 1.53 c | 6~7 |
| 9sr-25 | 3.72± 0.07 bc | 51.28± 0.77 c | 13.19± 0.39 ab | 63.21± 4.26 c | 4~5 |
| 9sr-14 | 3.41± 0.14 c | 41.63± 2.30 d | 9.72± 0.23 c | 61.63± 0.53 c | 4~5 |
| 9sr-3 | 2.77± 0.07 d | 33.33± 1.76 e | 9.36± 0.75 c | - | 4~5 |
| r9ox-19 | 4.02± 0.03 abc | 61.07± 0.91 ab | 14.12± 0.32 ab | 83.58± 3.77 a | 5~6 |
| r9ox-2 | 5.26± 1.13 a | 53.18± 5.44 bc | 14.81± 1.54 a | 78.81± 6.92 ab | 5~6 |
| r9ox-21 | 4.22± 0.68 ab | 56.27± 7.01 abc | 13.11± 1.27 ab | 82.88± 5.17a | 5~6 |

^*^ Measurements of the leaf length and width of the second leaves of the R3 stage tillers from top. The leaf blade width was measured at the position 1 cm from the base of the leaves. ^§^ The bottom internode diameters of the R3 stage tillers were measured with vernier caliper and statistically analyzed. ^&^ Internode number and rachis length were measured from the R3 stage tillers. The data are shown as the means of four biological replicates (with 20 technical repeats each) ± SD. Different letters in the same column represent significant differences (*P* < 0.05).

**Table S5.** Statistical analysis of the morphological parameters of WT and transgenic plants overexpressing *PvGRF1-SRDX* (1sr) and *rPvGRF1* (r1ox).

| Lines | Stem diameter (mm) ^§^ | Leaf length (cm) ^*^ | Blade width (mm) ^*^ | Richis length (cm) ^&^ | Internode number^&^ |
| --- | --- | --- | --- | --- | --- |
| WT | 3.66± 0.56 ed | 52.07± 4.75 ab | 13.53± 0.79 ab | 78.28± 8.98 bcd | 6~7 |
| 1sr-1 | 3.63± 0.67 ed | 50.87± 2.79 abc | 11.71± 0.49 de | 71.16± 4.27 d | 4~6 |
| 1sr-15 | 4.17± 0.64 cd | 41.74± 2.26 e | 11.32± 1.04 e | 73.12± 6.51 d | 4~6 |
| 1sr-8 | 2.81± 0.28 f | 45.80± 6.24 d | 10.94± 0.58 ef | 55.89± 6.42 e | 3~5 |
| r1ox-13 | 5.60± 0.87 a | 52.31± 3.92 ab | 13.88± 1.18 a | 86.05± 5.26 a | 6~8 |
| r1ox-11 | 4.36± 0.78 c | 51.66± 2.12 ab | 12.91± 1.32 bc | 84.36± 8.27 ab | 5~7 |
| r1ox-5 | 3.51± 0.59 e | 49.74± 3.46 bc | 11.73± 0.84 de | 83.11± 4.61 abc | 5~6 |

^*^ The leaf length and width of the second leaves of the R3 stage tillers from top were measured. The leaf blade width was measured at the position1 cm from the base of the leaves. ^§^ The bottom internode diameters of the R3 stage tillers were measured with vernier caliper and statistically analyzed. ^&^ Internode number and rachis length were measured from the R3 stage tillers. The data are shown as the means of four biological replicates (with 20 technical repeats each) ± SD. Different letters in the same column represent significant differences (*P* < 0.05).

**Table S6.** The morphological characteristics of the transgenic plants.

| Lines | Stem diameter (mm) ^§^ | Leaf length (cm) ^*^ | Blade width (mm) ^*^ | Rachis length (cm) ^&^ | Internode number^&^ | Internode length (cm) ^§^ |
| --- | --- | --- | --- | --- | --- | --- |
| WT | 5.21±1.04 abc | 75.00±6.66 a | 15.67±1.36 a | 93.05±6.12 a | 4-6 | 22.39±4.01 a |
| OE17 | 4.66± 0.86 cd | 41.39±4.58 f | 10.17±0.82 c | 9.13±3.32 f | 4-6 | 14.07±1.97 f |
| 9ox/OE17-L41 | 4.66±0.78 cd | 72.57±7.14 ab | 15.63±1.18 a | 76.75±22.70 b | 4-6 | 20.00±2.48 bc |
| 9ox/OE17-M27 | 5.72±0.85 a | 68.19±4.28 bc | 15.73±2.12 a | 73.42±3.77 b | 5-6 | 19.72±2.93 bc |
| 9ox/OE17-M19 | 5.54±0.65 a | 64.15±15.09 cd | 15.96±1.67 a | 61.52±14.56 c | 4-6 | 19.68±4.05 cd |
| 9ox/OE17-H6 | 5.53±0.63 a | 73.89±4.49 ab | 15.92±1.27 a | 80.15±11.86 b | 5-6 | 20.07±1.88 bc |
| r9ox/OE17-L4 | 4.84±0.49 bc | 57.72±7.69 e | 12.77±1.28 b | 31.71±7.93 e | 5-6 | 16.96±1.11 de |
| r9ox/OE17-M19 | 5.53±0.84 a | 60.93±4.62 de | 15.50±1.15 a | 46.22±12.06 d | 4-6 | 17.17±2.84 de |
| r9ox/OE17-M36 | 5.12±0.80 abc | 68.38±7.00 bc | 15.37±1.69 a | 53.65±7.38 c | 4-6 | 19.06±2.25 cd |
| r9ox/OE17-H48 | 5.34±0.71 ab | 71.58±4.72 ab | 16.18±1.17 a | 84.25±10.58 ab | 4-6 | 21.60±1.69 ab |

^*^ The leaf length and width of the second leaves of the R3 stage tillers from top were measured. The leaf blade width was measured at the position 1 cm from the base of the leaves. ^§^ The bottom internode diameters of the R3 stage tillers were measured and statistically analyzed. ^&^ Internode number, internode length and rachis length were measured from the R3 stage tillers. The data are shown as the means of four biological replicates (with 20 technical repeats each) ± SD. Different letters in the same column represent significant differences (*P* < 0.05).

**Table S7.** Cell wall enzymatic hydrolysis analysis of WT and transgenic plants overexpressing *PvGRF9-SRDX* (9sr) and *rPvGRF9* (r9ox).

| Lines | Unpretreated | | | Pretreated | | |
| --- | --- | --- | --- | --- | --- | --- |
|  | Glucose mg/g CWR | Xylose mg/g CWR | Carbohydrate yield mg/g CWR | Glucose mg/g CWR | Xylose mg/g CWR | Carbohydrate yield mg/g CWR |
| WT | 29.85±0.16b | 5.41±0013b | 35.26±0.28c | 44.42±1.47bc | 39.44±1.24c | 83.86±0.23bc |
| 9sr-22 | 35.12±0.31a | 4.88±0.42bc | 40.00±0.11ab | 47.26±0.76ab | 38.96±0.57c | 86.22±0.19b |
| 9sr-25 | 31.80±0.93b | 6.41±0.21a | 38.21±0.72bc | 47.25±0.13ab | 49.66±1.95a | 96.91±1.82a |
| 9sr-14 | 37.31±0.13a | 5.32±0.08b | 42.63±0.04a | 49.51±0.12a | 47.21±0.38ab | 96.72±0.50a |
| r9ox-19 | 23.77±0.18d | 5.01±0.10bc | 28.78±0.08d | 41.47±1.57cd | 40.00±2.91bc | 81.47±1.35c |
| r9ox-2 | 20.00±2.29e | 4.14±0.54d | 24.14±2.83e | 44.83±2.46b | 42.4±6.84abc | 87.23±4.39b |
| r9ox-21 | 26.90±1.33c | 4.39±0.21cd | 31.29±1.54d | 41.00±0.14d | 35.37±0.95c | 76.37±0.81d |

The data are shown as the means of three biological replicates (with five technical repeats each) ± SD. The different letters in the same column indicate statistically significant differences (*P* < 0.05).

**Table S8.** A list of primers used in this study.

| **Primer name** | **Primer sequence** |
| --- | --- |
| **Primers for cloning** | |
| 396a (OX)_XbaF | TCTAGATTTCCTTTCGATAGCGGTGCA |
| 396a (OX)_SalR | GTCGACACCAATATACCAACAAACATTACAT |
| PvGRF9 (SDRX)_XbaF | TCTAGAATGAGTGCCACCGAGTTCCG |
| PvGRF9 (SDRX)_BamR | GGATCCTGGTGAATGGTCAAGATTCAA |
| GRF9(OX)_XbaF | TCTAGAATGAGTGCCACCGAGTTCCG |
| GRF9(OX)_KpnR | GGTACCTCATGGTGAATGGTCAAGATTCAA |
| rGRF9_R | GTTCTCGACTGGTTTCCTAGAACGATGACGATTTCTGTTGATGTGCC |
| rGRF9_F | CGTTCTAGGAAACCAGTCGAGAACCAACCTAAAA AGACCGCCAA AG |
| PvGRF1 (SDRX)_XbaF | TCTAGAATGATGATGATGAGCCGTCACGC |
| PvGRF1 (SDRX)_BamR | GGATCCCTCATCATTACGGTAGCGGGAG |
| GRF1(OX)_XbaF | TCTAGAATGATGATGATGAGCCGTCACGC |
| GRF1(OX)_KpnR | GGTACCTCACTCATCATTACGGTAGCGGGAG |
| rGRF1_R | CATCTCGACTGGTTTCCTAGAACGGTTCTTGCCGCGGTGCATGTGCTT |
| rGRF1_F | TTCTAGGAAACCAGTCGAGATGTCCCTTGCCACGCCGGCGCC |
| PvGRF3 (SDRX)_XbaF | TCTAGAATGGCGATGCCCTTTGCCT |
| PvGRF3 (SDRX)_SalR | GTCGACTTACGCAAAGCCCAGGCGCAGTTCCAGATCCAGATCCAGGGATCCGCTCTGAGACGTTGGGAT |
| GRF3(OX)_XbaF | TCTAGAATGGCGATGCCCTTTGCCT |
| GRF3(OX)_KpnR | GGTACCTCAGCTCTGAGACGTTGGGAT |
| GRF3(IN)_R | CGCCTCGACTGGTTTCCTAGAACGGTTGCGGCCGCGGTGCATGTGGCG |
| GRF3(IN)_F | TTCTAGGAAACCAGTCGAGGCGCAGCTCGTCGCCCAGCAGC |
| **Primers for genomic DNA PCR and RT-PCR** | |
| Os396_F | CGCATGATGAATAATCCCTTTG |
| Os396_R | GCTGGCGGCGTCACTTACA |
| 35S_F | CGCACAATCCCACTATCCTTC |
| PvGRF9_R | TCATGGTGAATGGTCAAGATTCAA |
| **Primers for testing mature miR396** | |
| stem-loop miR396a | GTCGTATCCAGTGCAGGGTCCGAGGTATTCGCACTGGATACGACCAGTTC |
| 396a-Forward | CGGCGGTTCCACAGCTTTCTT |

**Table S8.** Continued.

| **Primer name** | **Primer sequence** |
| --- | --- |
| **Primers for reference genes** | |
| U6_F | AGGAGCCGCTGGATCTGATA |
| U6_R | CGACCTCCTCAACATCTCCA |
| Ubq_F | CAGCGAGGGCTCAATAATTCCA |
| Ubq_R | TCTGGCGGACTACAATATCCA |
| **Primers for lignin biosynthesis genes and GA biosynthesis and transduction genes** | |
| PvGA20ox-2F | ACCCTCTCCTTCGGCTTCCAC |
| PvGA20ox-2R | GCCTTCATCTCCTCGCAGTACC |
| PvGA20ox-3F | TCGCTCACCATCCTCCACCAG |
| PvGA20ox-3R | CGCCGATGTTGACGACCAAGG |
| PvGA2ox-3F | ACCGACCCGCAGCTCATCTC |
| PvGA2ox-3R | CGCCGACGTTGACGAAGAAGG |
| PvGRAS_F | GACTGTTCTGATGGCTCCTGATGC |
| PvGRAS_R | TGTGCGGACAATTCCTGGTGATG |
| PvGID1_F | CCAAGAGCCTCATCATCGTGTCG |
| PvGID1_R | TCCTCCATGACCTCGTGGTAGTG |
| PvGID2_F | CTGGTGTTCGAGGTGCTGATGC |
| PvGID2_R | CGCCGAACGAGAGCACAACC |
| PvF5H_F | GGCGAGGACCAGGAGGAGTTC |
| PvF5H_R | GGGTGCGGCGGTTGATGC |
| PvCOMT_F | CCTGCCGATGACGCTCAAGAAC |
| PvCOMT_R | CCGCGCCACCACCTCCTC |
| PvCCR_F | GCGTCGTGGCTCGTCAA |
| PvCCR_R | TCGGGTCATCTGGGTTCCT |
| PvPAL_F | ACGGCGGCGACATCTACGG |
| PvPAL_R | TCACCTGAGCAGCTCGACCTG |
| PvC4H_F | GGGCAGTTCAGCAACCAGAT |
| PvC4H_R | CGCGTTTCCGGGACTCTAG |
| PvC3H_F | CGTGAACAATGGGATCAGGATAG |
| PvC3H_R | GCGGACACAACCATCTCAAATAC |
| Pv4CL_F | CGAGCAGATCATGAAAGGTTACC |
| Pv4CL_R | CAGCCAGCCGTCCTTGTC |
| **Primers for cellulose and hemicellulose synthetic genes** | |
| PvCESA1_F | GCATCCAGGGTCCAGTTTATGTG |
| PvCESA1_R | CCAGATCGGCTTCGGTCAATAC |
| PvCESA3_F | GCATTTCCCTCCTCGTCGTC |
| PvCESA3_R | ACTTGCTCCTCTCGCTCTAGC |
| PvCSLA6_F | TGCACATTACGGAGCTTGGTG |
| PvCSLA6_R | TGGTCCCTCCCATACGCAAG |
| PvCSLC2_F | GTAGAAGCAGCCAAGGCACTG |
| PvCSLC2_R | GAGTCGCTGTACGCCTCTTG |
| PvIRX10_F | GCCAAGGATTCTAGATGCCTCAGC |
| PvIRX10_R | GGTCGCATGTCGTGTATACTGGAG |
| PvIRX9_F | AACAATGTCGGCAGGCGAGAAG |
| PvIRX9_R | CCAGCAGCACCAGCAGGATTC |
| PvIRX14_F | CTGCTGCCTCATCTCGCTCTTC |
| PvIRX14_R | GTCGTGGTCGTGGTGGTTGTG |
| **Primers for Auxin biosynthesis and signaling genes** | |
| PvIAA30_F | GCGGCGTTCGTGAAGGTGAG |
| PvIAA30_R | CTCCTGGTAGCTCCCGTACATCC |
| PvYUCC2_F | GTGGAGCGAAAGTGAGAGCAAGAG |
| PvYUCC2_R | TCCGAGCGAGCAACAACAGAATC |
| PvTAA1_F | CGGGAGCGGTAAGATTCAGAAGC |
| PvTAA1_R | TATGGCGACGGAGACGAAGAGAG |
| PvTIR1_F | TGCTTTCAATCGCCTTCGCT |
| PvTIR1_R | AAATGGGCAGTCCCTGATCTCC |
| PvAFB1_F | TGCTGGTTGTAAAAGCCTAAGAA |
| PvAFB1_R | ATCCATCACCCATAAGAGTTGC |
| PvARF_F | TGCAGCAGAGCGGTGGAGTC |
| PvARF_R | GGTTCAAGTAGCAGCAGGGCATAG |

**Table S8.** continued.

| **Primer name** | **Primer sequence** | **Reference** |
| --- | --- | --- |
| **Primers for PvGRFs qRT-PCR** | |  |
| pvGRF8_F | GCCGAGATGGGTTCTTTGG | - |
| pvGRF8_R | ACTGCGTCGGAGTGAATGG | - |
| pvGRF6_F | CAAGAACGGTAACGGCGAAG | - |
| pvGRF6_R | GGATGCTCGGATGGTTTACTACA | - |
| PvGRF1_F | CGCACGAGAAATCAAACAAGG | - |
| PvGRF1_R | CATCATTACGGTAGCGGGAG | - |
| PvGRF4_F | AAAACTCCGCATTTCCTCTCTC | - |
| PvGRF4_R | AGGGGCTGCCTGTCCATCTT | - |
| PvGRF9_F | CAGAACCTGGAAGATGCCGT | - |
| PvGRF9_R | GCTTTCTTGAACGATGACGATT | - |
| PvGRF3_F | ATGAGGTCAGCACCAGAGAGTCGT | - |
| PvGRF3_R | CGAGCGGGAATACCATCAAG | - |
| PvGRF5_F | CCCCGAACTATTTCTTTCACTG | - |
| PvGRF5_R | GCACACAAACACTTGCATCATC | - |
| PvGRF2_F | GTCCTCCTCTATCCTCCGTTGG | - |
| PvGRF2_R | TGCCCGCTCAGCATCATCTC | - |
| PvGRF10_F | GACCACAGTTGCCGTTGCTT | - |
| PvGRF10_R | TGCTGCGAGTCGGTATCCTT | - |
| PvGRF11_F | TGCCTACTCATCTCGTCTTTCC | - |
| PvGRF11_R | AGCATCGCCACTTTTTACCAT | - |
| **RNA adaptor and primers for 5'RLM RACE** | | |
| RNA 5’ adaptor | CGACUGGAGCACAGGACACUGACAUGGACUGAAGGAGUAGAAA | Wang and Fang, 2015 |
| Oligo(dT)_30_ primer | ATTCTAGAGGCCGAGGCGGCCGACATG-d(T) 30 |  |
| GeneRacer 5′ primer | AGGACACTGACATGGACTGAAGGAGTAG |  |
| Universal primers | ATTCTAGAGGCCGAGGCGGCCGACATG |  |
| GRF9_GSP1 | TTTCTCTTTGCTCAACATTGTCCTG | - |
| GRF1-GSP1 | GGGACGAGGTCGTGGTGGCGT | - |
| GRF3-GSP1 | GCCTGCACGGACGCCGTTGC | - |
| GRF2-GSP1 | CCATGCTGGGGTTGCTGCTGGT | - |
| GRF4-GSP1 | AGTGGTTCTGGAAGCTGCTGCCG | - |
| GRF5-GSP1 | TTACCCCCCACGGTGAGGGAGTC | - |
| GRF6-GSP1 | CTGGTGCTGACTGACAGTGAGGCC | - |
| GRF8-GSP1 | CACCTGCTGCTGCCAAGGCAC | - |
| GRF10-GSP1 | GGAGAAGCCGAGCCCGTGCG | - |

-, Initially designed in this work.


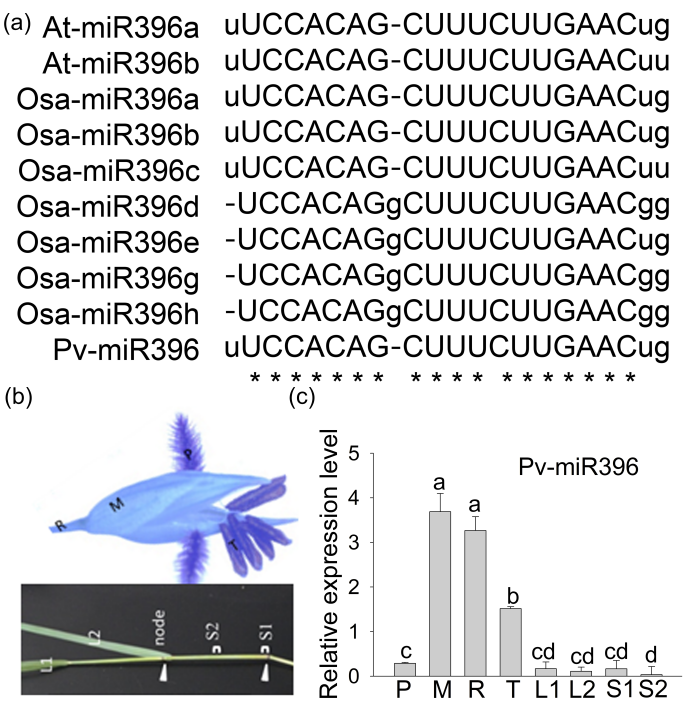


**Figure S1.** The sequence alignment and expression pattern of miR396. (a) The sequence alignment of miR396 family of *Arabidopsis* (At), rice (Osa) and switchgrass (Pv). (b) The structure of a typical switchgrass flower (top) and a representative E3 stage tiller showing the sampling parts (bottom); R, rachilla; M, lemma; P, pistil; T, stamen; stem (S1, S2); leaf (L1, L2); S1 and S2 were sampled from the second internode of the E3 stage tiller; L1 and L2 were sampled at the position 1 cm from the base of the leaves. (c) The relative expression level of miR396 in different tissues of switchgrass (n=3). The error bar indicates standard deviation. The different letters indicate statistically significant differences determined by Duncan’s multiple range test (*P* < 0.05).


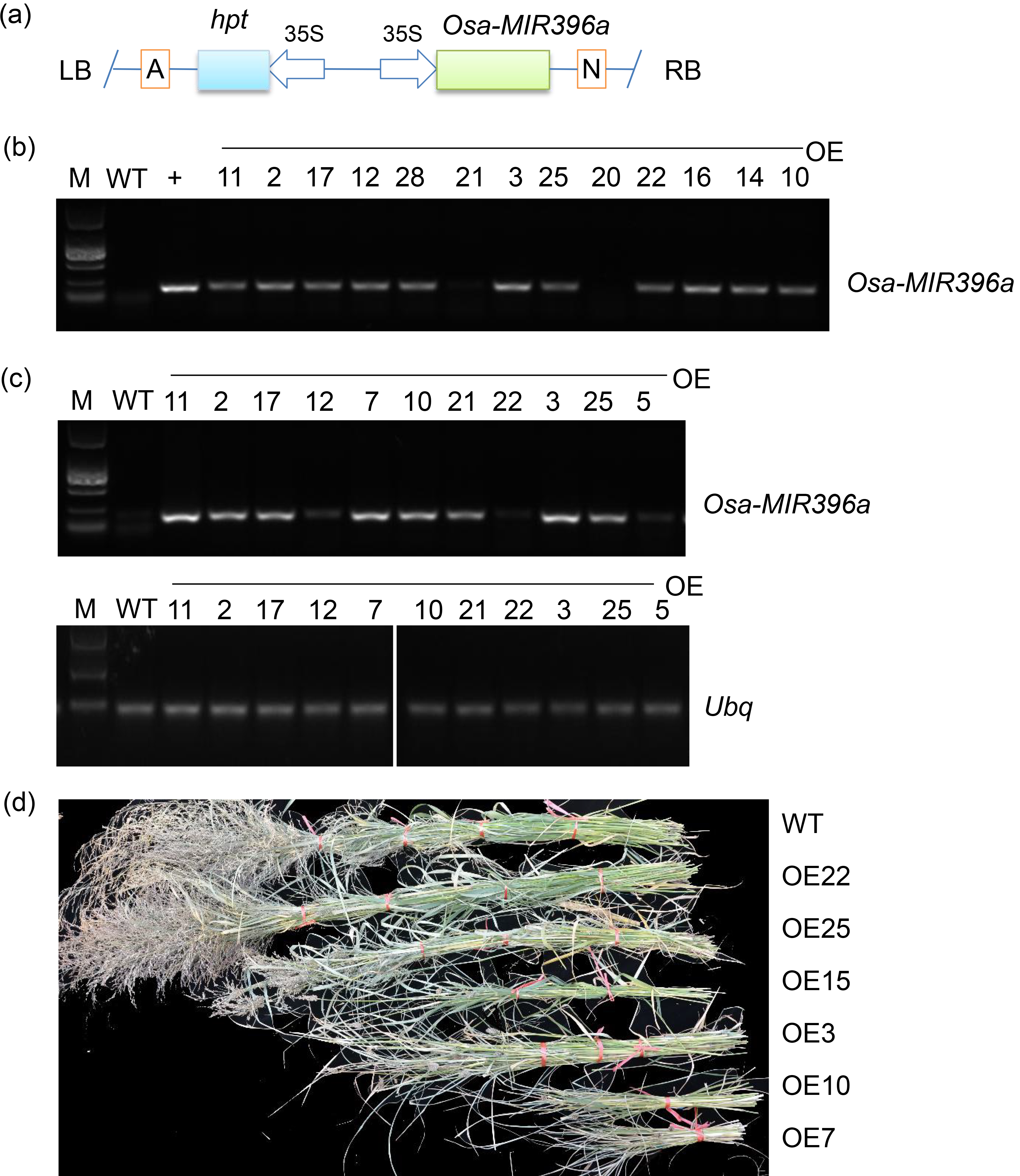


**Figure S2.** Production of the *Osa-MIR396a* transgenic switchgrass plants. (a) The schematic T-DNA region of the vectors for overexpression of *Osa-MIR396a*; LB, left border; 35S, CaMV 35S promoter; hpt, hygromycin B resistance gene; A, CaMV 35S polyA terminator; N, nopaline synthase gene terminator; RB, right border. (b) *Osa-MIR396a* insertion in OE plants revealed by PCR; M: DNA marker; +: positive control. (c) *Osa-MIR396a* expression in OE plants revealed by RT-PCR using *ubiquitin* （*Ubq*） gene as an internal control. (d) Morphological characteristics of the OE plants.


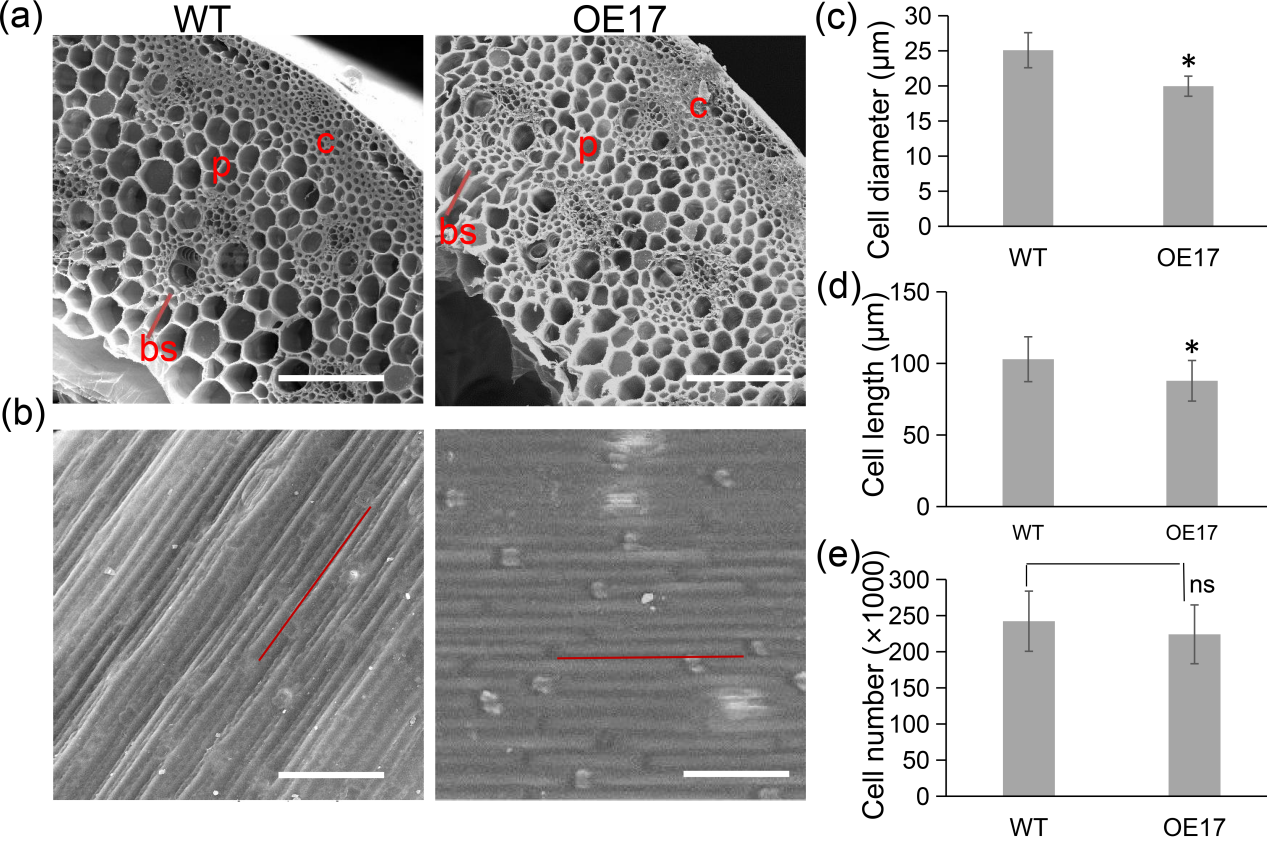


**Figure S3.** Scanning electron microscopy of the middle part of the first internode (top) cross section and epidermal cells of the E3 stage tiller (the first internode from the top, 1NE3). (a) Comparison of the cross sections of the WT and OE17. The scale bar =100 μm; bs, vascular bundle sheath cells; p, parenchyma; c, collenchyma. (b) Comparison of the epidermal cells; scale bar = 50 μm. The red line represents a long cell as an example. (c) Comparison of the diameters of the parenchymal cells (n=80). (d) Comparison of the length of the long cell in the 1NE3 surface (n=50). The stars indicate statistically significant differences determined by Duncan’s multiple range test (*P* < 0.05). (e) Comparison of the long cell number (internode length/ long-cell length) of 1NE3 internode (n=50). ns, no significant difference.


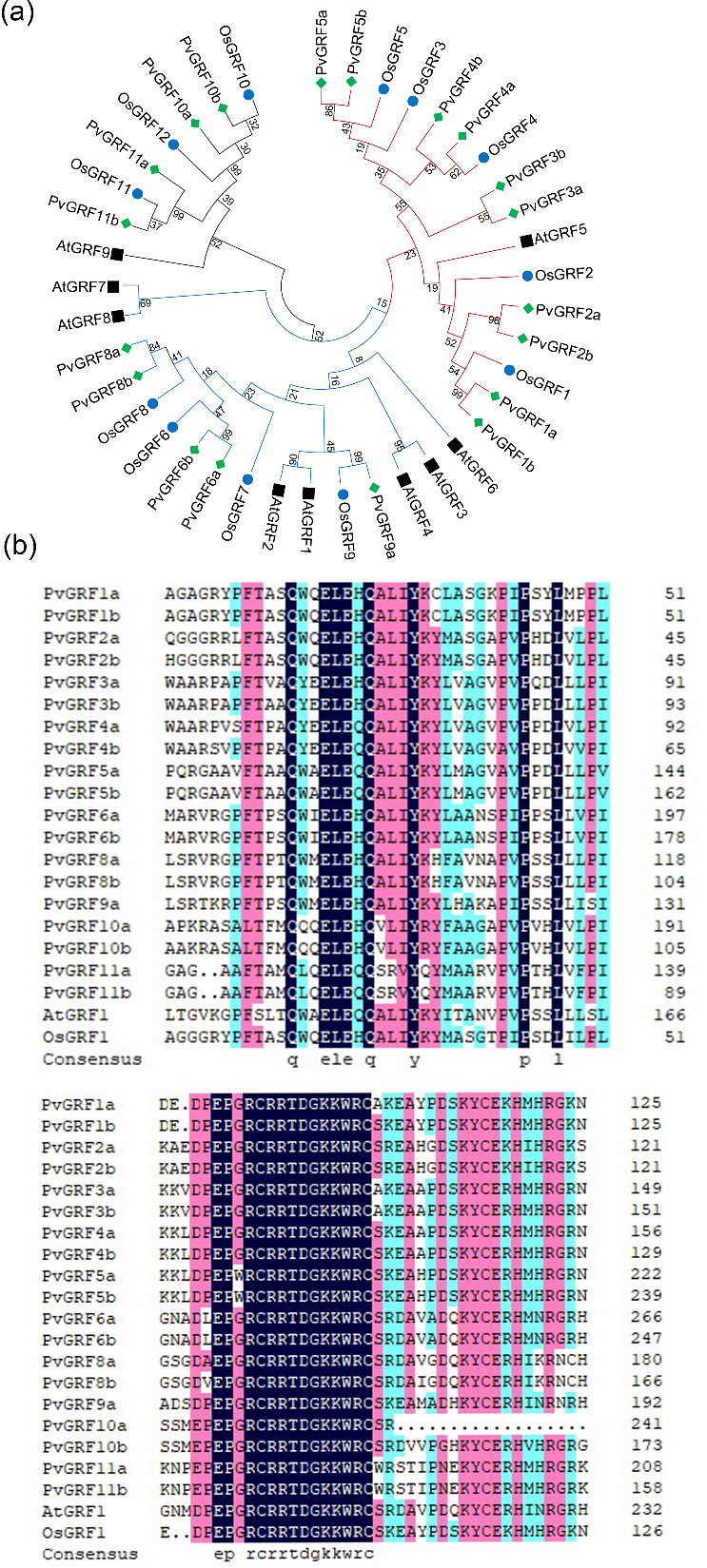


**Figure S4.** Sequence alignment of the GRF proteins of switchgrass, rice and *Arabidopsis*. (a) The phylogenetic tree of PvGRFs, OsGRFs and AtGRFs. (b) The QLQ and WRC domains of the switchgrass PvGRFs, *Arabidopsis* AtGRF1 and rice OsGRF1.


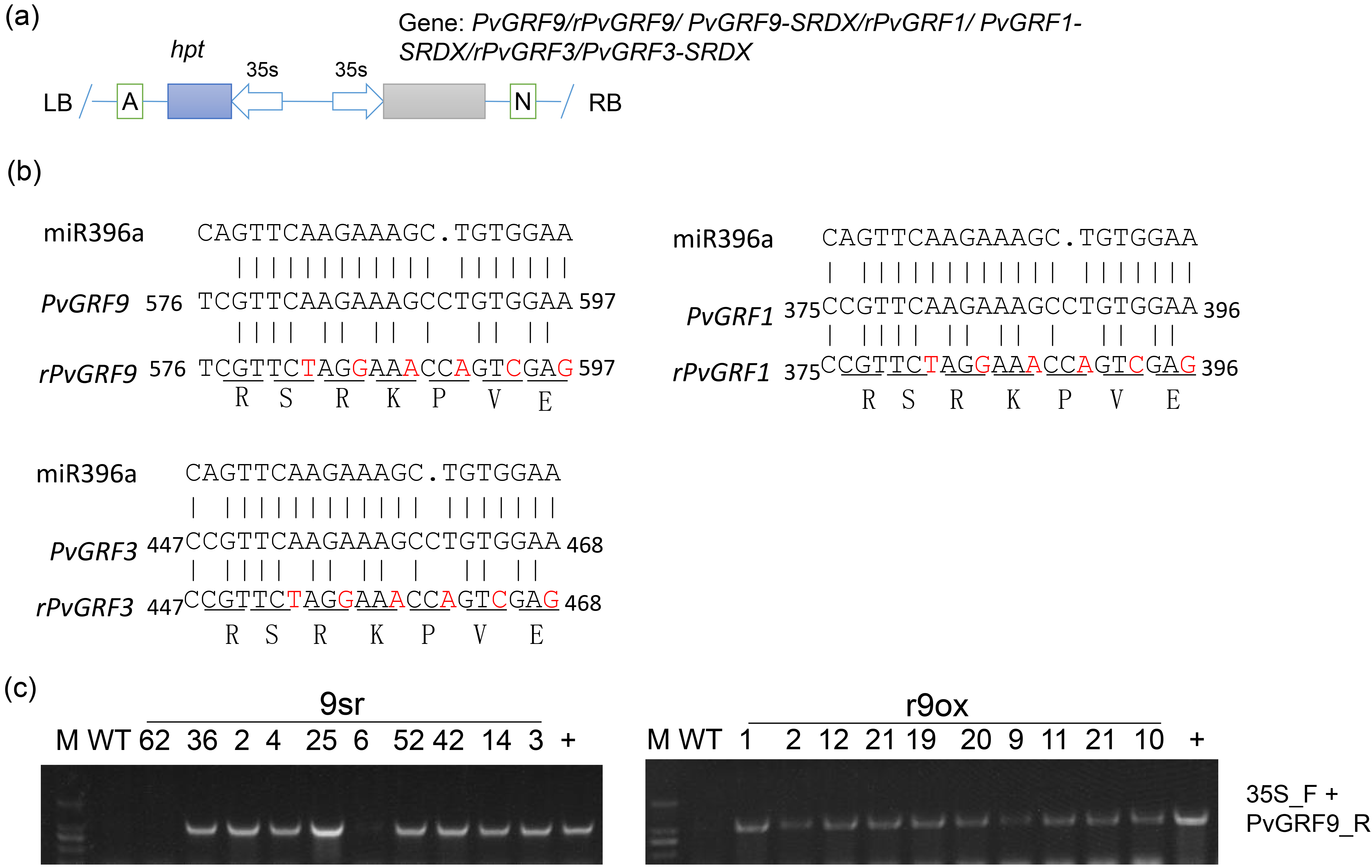


**Figure S5.** The schematic map of the *PvGRFs*-related gene constructions and PCR analysis of *PvGRF9*-related genes in transgenic plants. (a) The schematic map of the *PvGRFs*-related gene expression vectors. LB, left border; 35S, CaMV 35S promoter; hpt, hygromycin B resistance gene; A, CaMV 35S polyA terminator; N, nopaline synthase gene terminator; RB, right border; *rPvGRF1/3/9*, synonymous mutation of *PvGRF1/3/9,* in which miR396 target site was abolished; *PvGRF1/3/9-SRDX*, the 3’ ends of *PvGRF1/3/9* linked to a sequence encoding the SRDX domain. (b) The mutant bases of *rPvGRF9*, *rPvGRF1* and *rPvGRF3* were shown in red color. (c) PCR analysis of the *PvGRF9-SRDX* (9sr) and *rPvGRF9* (r9ox) overexpression transgenic plants. M: DNA marker; +: positive control.

**
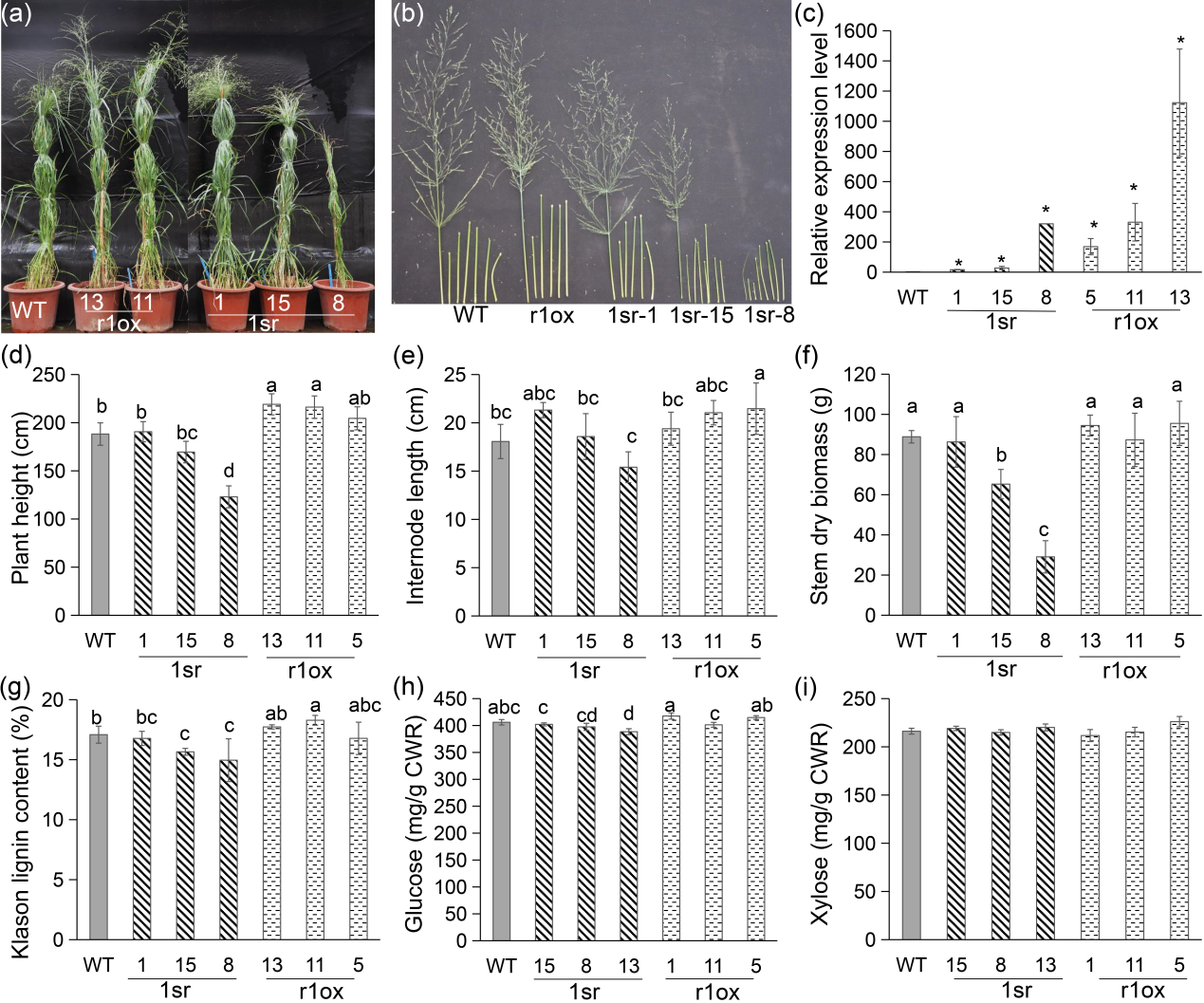
Figure S6.** *PvGRF1* positively regulates plant height and lignin content. (a) The morphological characteristics of WT, *PvGRF1-SRDX* (1sr) overexpressing plants and *rPvGRF1* (r1ox) overexpressing plants. (b) A typical R3 stage stem of the WT, 1sr and r1ox plants. (c) *PvGRF1* expression levels of the WT, 9sr and r9ox lines (n=3). Asterisks indicate statistically significant differences compared with WT (*P* < 0.05). (d) Comparison of the plant height and internode length (e) of the WT, 1sr and r1ox lines (n=4) with twenty technical repeats each. (f) Stem dry biomass of the WT, 1sr and r1ox lines (n=4). The Klason lignin content (g), glucose content (h) and xylose content (i) of the stem cell wall residues of the WT, 1sr and r1ox lines (n=3) with five technical repeats each. The error bar indicates standard deviation. The different letters indicate statistically significant differences determined by Duncan’s multiple range test (*P* < 0.05).


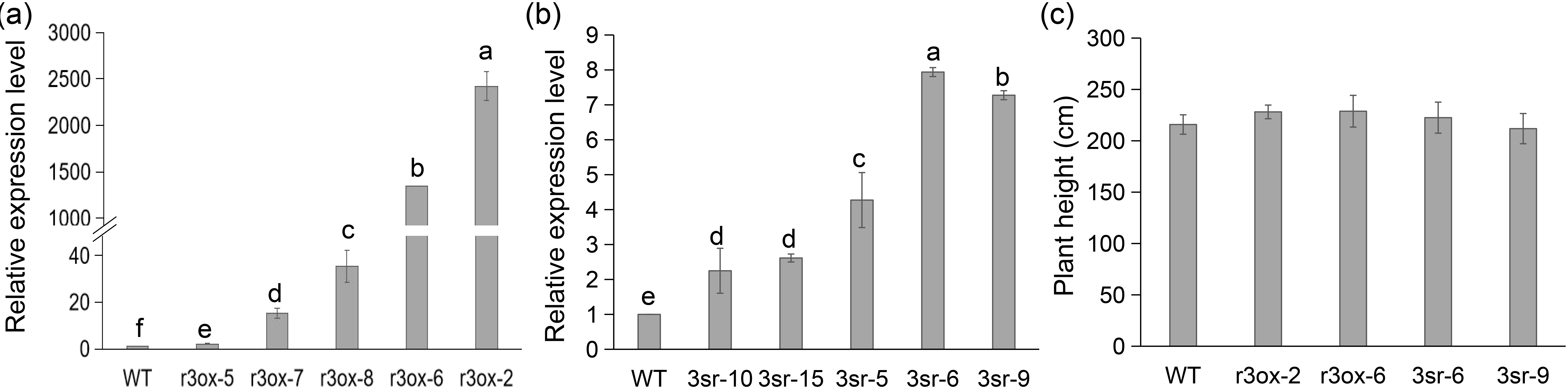


**Figure S7.** *PvGRF3* showed no significant effect on switchgrass plant height. The qRT-PCR analysis of *rPvGRF3* (r3ox) (a) and *PvGRF3-SRDX* (3sr) overexpression in transgenic plants (b) (n=3). (c) Comparison of the plant height of the WT, r3ox, and 3sr lines (n=4) with twenty technical repeats each. The error bar indicates standard deviation. The different letters indicate statistically significant differences determined by Duncan’s multiple range test (*P* < 0.05).


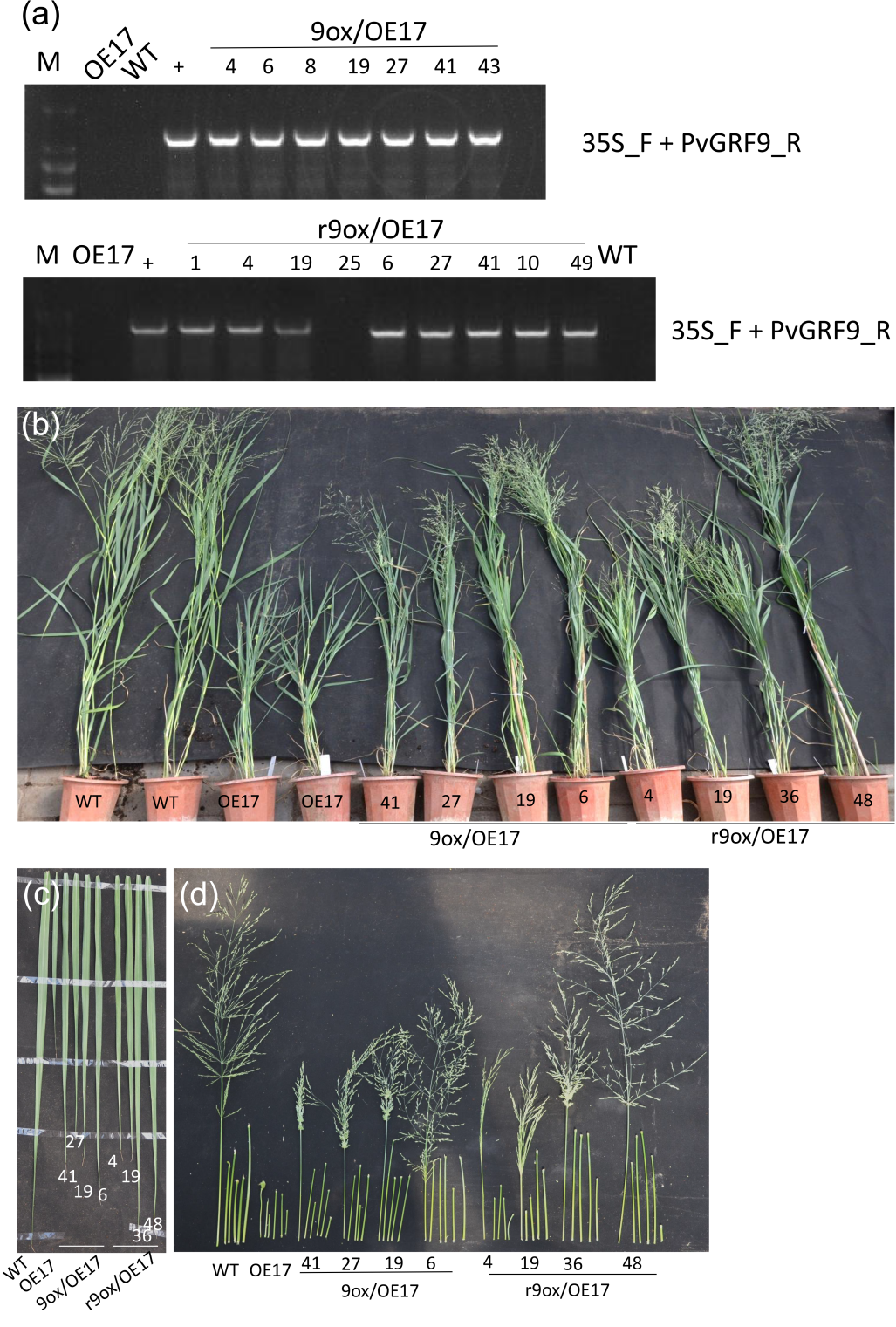


**Figure S8.** Transgenic plants verification by PCR tests (a) and the morphological characteristics comparison with wild type (WT) and complementation OE17 plants. Comparison of the phenotype of the WT, OE17, 9ox/OE17 and r9ox/OE17 plants (b), leaf-shape (c), inflorescence and internode (d).

**
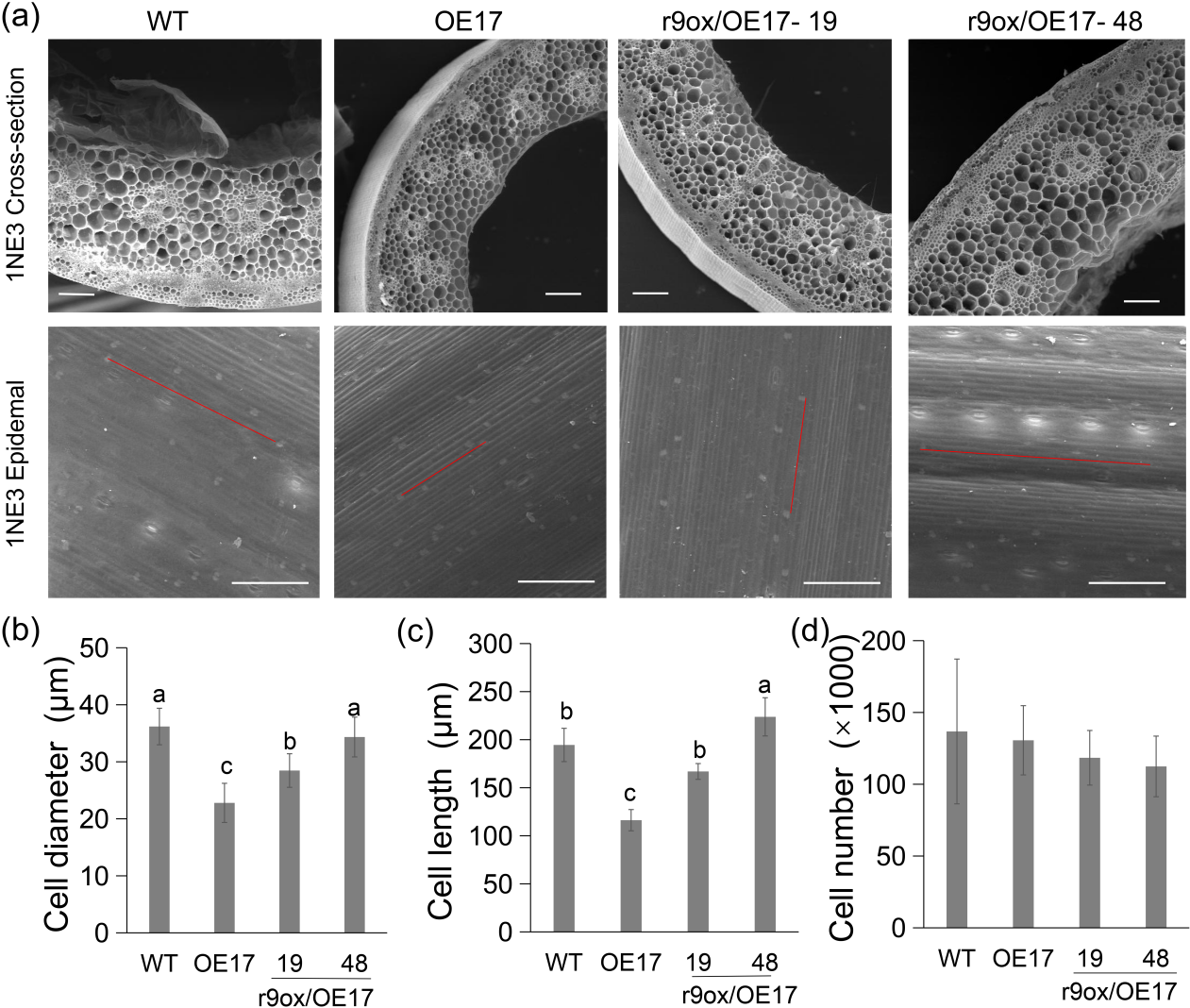
**

**Figure S9.** Scanning electron microscopy of the middle part of the first internode (top) cross section and epidermal cells, leaf and leaf sheath of the E3 stage tiller (the first internode from the top, 1NE3). (a) Comparison of the cross sections (scale bar =50 μm) and epidermal cells (scale bar =100 μm) of 1NE3. (b) Comparison of the diameters of parenchymal cells (n=80). (c) Comparison of the length of the long cell of 1NE3 surface (n=50). (d) The long cell number per internode (n=50). The different letters indicate statistically significant differences (*P* < 0.05).
